# Identification of in vivo roles of ErbB4-JMa and its direct nuclear signaling using a novel isoform-specific knock out mouse

**DOI:** 10.1038/s41598-022-21598-2

**Published:** 2022-10-14

**Authors:** Robert Doherty, Brenna L. MacLeod, Megan M. Nelson, Mostafa M. H. Ibrahim, Beatriz C. Borges, Nada W. Jaradat, Matthew C. Finneran, Roman J. Giger, Gabriel Corfas

**Affiliations:** 1 Department of Otolaryngology-Head and Neck Surgery, Kresge Hearing Research Institute, University of Michigan Medical School, Medical Sciences I Building, Rm. 5428, 1150 West Medical Center Drive, Ann Arbor, MI 48109-5616 USA; 2 Neuroscience Graduate Program, University of Michigan Medical School, Ann Arbor, MI 48109 USA; 3 Department of Cell and Developmental Biology, University of Michigan Medical School, Ann Arbor, MI 48109 USA

**Keywords:** Cell signalling, Developmental biology, Neuroscience

## Abstract

Like all receptor tyrosine kinases (RTKs), ErbB4 signals through a canonical signaling involving phosphorylation cascades. However, ErbB4 can also signal through a non-canonical mechanism whereby the intracellular domain is released into the cytoplasm by regulated intramembrane proteolysis (RIP) and translocates to the nucleus where it regulates transcription. These different signaling mechanisms depend on the generation of alternative spliced isoforms, a RIP cleavable ErbB4-JMa and an uncleavable ErbB4-JMb. Non-canonical signaling by ErbB4-JMa has been implicated in the regulation of brain, heart, mammary gland, lung, and immune cell development. However, most studies on non-canonical ErbB4 signaling have been performed in vitro due to the lack of an adequate mouse model. We created an ErbB4-JMa specific knock out mouse and demonstrate that RIP-dependent, non-canonical signaling by ErbB4-JMa is required for the regulation of GFAP expression during cortical development. We also show that ErbB4-JMa signaling is not required for the development of the heart, mammary glands, sensory ganglia. Furthermore, we identify genes whose expression during cortical development is regulated by ErbB4, and show that the expression of three of them, *CRYM*, *PRSS12* and *DBi*, depend on ErbB4-JMa whereas *WDFY1* relies on ErbB4-JMb. Thus, we provide the first animal model to directly study the roles of ErbB4-JMa and non-canonical ErbB4 signaling in vivo.

## Introduction

The receptor tyrosine kinase (RTK) family consists of 58 single-pass transmembrane receptors that mediate the actions of growth factors, hormones, and cytokines to regulate numerous biological processes^1^. RTKs play critical roles in many cellular developmental and physiological functions and their dysregulation is involved in numerous diseases and disorders^1,2^. Initially, it was believed that RTKs signal through a single mechanism, now called the canonical RTK pathway. Briefly, ligand binding induces receptor dimerization resulting in RTK trans-phosphorylation, activation of adaptor proteins and phosphorylation of intracellular kinases to regulate cellular growth, survival, migration, and transcription^1^. It was later discovered that the ErbB4 RTK, a member of the EGFR family, is also capable of signaling through a non-canonical mechanism in which the intracellular domain (ICD) is released by regulated intramembrane proteolysis (RIP) and translocates to the nucleus where it regulates transcription^3–5^.

ErbB4 is activated by several cognate ligands, including NRG1, betacellulin and HB-EGF^6,7^. Alternative splicing in the extracellular juxtamembrane domain (EJD) generates two ErbB4 isoforms that differ in their ability to perform direct nuclear signaling^8^. The EJD of ErbB4-JMa isoform, which is encoded by exon 16a, contains a cleavage site for tumor necrosis factor-α-converting enzyme (TACE), whereas the EJD of the ErbB4-JMb isoform, encoded by exon 16b, is uncleavable^8,9^. Activation of ErbB4-JMa by its ligand neuregulin 1 (NRG1)^10^ or activation of protein kinase C by 2-O-tetradecanoylphorbol-13-acetate (TPA)^5,8,9,11^ promote TACE-dependent cleavage in the EJD, generating a soluble extracellular domain and a membrane-tethered 80-kD intracellular domain fragment (mE4ICD). Then, mE4ICD can be cleaved by presenilin/γ-secretase complex (PS1) in the intramembrane domain, releasing the soluble intracellular domain (E4ICD)^3,5,9^. Activated E4ICD can shuttle to the nucleus via a nuclear localization sequence where it interacts with other nuclear proteins and chromatin to induce transcriptional changes in gene expression^3,12^. While direct nuclear signaling by RTKs was initially thought to be rare, it has been suggested that over half RTKs might be capable of signaling through this type of non-canonical mechanism^13,14^.

ErbB4 is important for the development and function of several organs including the heart^15^, mammary glands^16^, lungs^17^, and nervous system^6^. ErbB4 regulates several neurodevelopmental processes such as neuronal migration^18–20^, differentiation^3^, axon guidance^21,22^, and synapse formation^23–25^. ErbB4 also regulates aspects of neuronal function and plasticity, including interneuron signaling^23,26^, glutamatergic and dopaminergic neurotransmission^24,27^, and hippocampal potentiation^28,29^. This receptor has also been implicated in nervous system disorders, including schizophrenia^6^, neurological injury repair^30^, and neurodegenerative diseases such as amyotrophic lateral sclerosis and Alzheimer’s disease^31,32^. However, the biological roles of ErbB4-JMa direct nuclear signaling have been tested only in a few processes. For example, our lab provided evidence that nuclear signaling by E4ICD is important for the timing of astrogenesis during cortical development^3^. Briefly, we showed that ligand induced cleavage of ErbB4-JMa in neural precursor cells (NPCs) leads to the formation of E4ICD which associates with TAB2 and NCoR to repress astrocytic gene expression by directly interacting with the promoters of the glial genes GFAP and S100β^3^. This conclusion was supported by the observation that astrogenesis occurs precociously in ErbB4^−/−^ mice, a defect that is rescued by transfection with a constitutively active E4ICD^3^. However, definitive formal proof for this has been lacking due to the absence of a ErbB4-JM KO mouse model. Non-canonical E4ICD signaling has also been proposed to play roles in other biological processes such as mammary gland^33^, lung^34^ and cardiac^35^ development. However, since these studies were performed using cells in culture and/or mice with total ErbB4 loss of function, evidence that ErbB4-JMa direct nuclear signaling regulates these processes is not available. Whereas mouse lines with defects in TACE or PS1 function exist^36–39^, since these enzymes have many critical biological roles, their phenotypes cannot be directly linked to ErbB4-JMa non-canonical signaling.

To fill these gaps in knowledge, we aimed to create mutant mice using CRISPR/Cas9 gene editing to eliminate TACE-mediated ErbB4-JMa cleavage and block E4ICD formation and hence non-canonical signaling. Unexpectedly, mutations that eliminate ErbB4 cleavage in heterologous cells failed to prevent E4ICD formation in vivo. Fortunately, the CRISPR/Cas9-mediated mutagenesis also created mice carrying a premature stop codon within ErbB4 Exon 16a, thus abolishing expression of ErbB4-JMa without altering ErbB4-JMb. Using this mutant line named ErbB4-JMa^−/−^, we now formally show that non-canonical ErbB4-JMa signaling is required for the regulation of GFAP expression by NPCs in vitro and in the cortical ventricular zone of the neonatal brain. Furthermore, phenotypic analysis also provides evidence that non-canonical signaling by ErbB4-JMa is not necessary for heart, mammary gland, and neural ganglia development. Moreover, using a combination of RNAseq and qRT-PCR, we identified 20 new genes whose expression in the embryonic brain is regulated by ErbB4, some requiring ErbB4-JMa, others not. In summary, we created a new mouse model and used it to test established hypotheses about the roles of canonical and non-canonical ErbB4 signaling in vivo and to discover new roles for ErbB4 in the regulation of gene expression in the developing brain. This mouse line also serves as proof of concept for an approach to study non-canonical RTK signaling in vivo.

## Results

### Mutation of the putative ErbB4-JMa TACE cleavage site prevents E4ICD formation in heterologous cells

**Figure 1:**
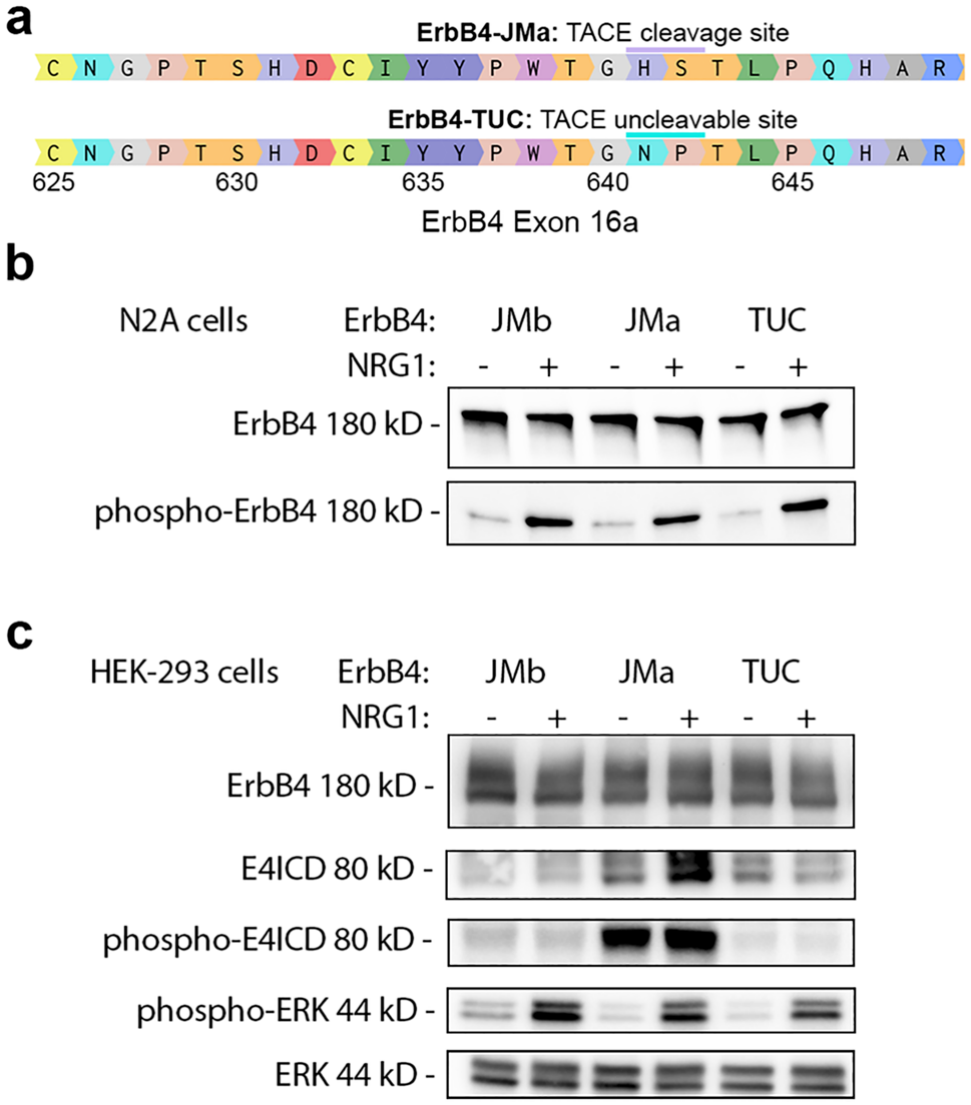
Mutation of the putative TACE cleavage site of ErbB4-JMa prevents E4ICD formation without altering RTK signaling in transfected cells. (**a**) Diagram of ErbB4 Exon 16a. ErbB4-JMa TACE cleavage sites (purple bar) and the H641N;S642P mutation sites to create the TACE-uncleavable (TUC) plasmid (teal bar) are shown. (**b**) Western blot analysis of N2A cells transfected with ErbB4-JMb, ErbB4-JMa, or ErbB4-TUC shows that TUC mutations do not affect NRG1-induced ErbB4 tyrosine phosphorylation. (**c**) Western blot analysis of HEK-293 cells transfected with ErbB4-JMb, ErbB4-JMa, or ErbB4-TUC shows the TUC mutations prevent the NRG1-induced formation of E4ICD seen in ErbB4-JMa, but do not affect the NRG1-induced ERK phosphorylation. The image depicting the 80 kD E4ICD band was obtained with a longer exposure than the image depicting the 180 kD full-length ErbB4.

To create a mouse mutant in which E4ICD direct nuclear signaling is eliminated, we first sought to identify a mutation that blocks TACE-dependent ErbB4-JMa cleavage. Previous studies pointed to H641 and S642 in exon 16a as key amino acids for this cleavage event^40^. Therefore, we used site-directed mutagenesis to create a full-length ErbB4 carrying H641N;S642P mutations, which we called ErbB4-TUC for “TACE-uncleavable ErbB4-JMa” (Fig. 1a). Analysis using transfection into cell lines that do not express ErbB4 showed that these mutations do not alter ErbB4’s ligand-induced autophosphorylation (Fig. 1b) and ERK activation (Fig. 1c). In contrast, ligand-induced formation of the 80 kD E4ICD band was absent in ErbB4-TUC expressing cells (Fig. 1c). These results suggested that introducing the H641N;S642P mutations into exon 16a of the *ERBB4* gene could create mice in which ErbB4-JMa behaves like ErbB4-JMb, retaining the capacity for canonical signaling but not E4ICD-mediated direct nuclear signaling.

### Creation of mice deficient in non-canonical ErbB4-JMa signaling using CRISPR/Cas9 gene editing

**Figure 2:**
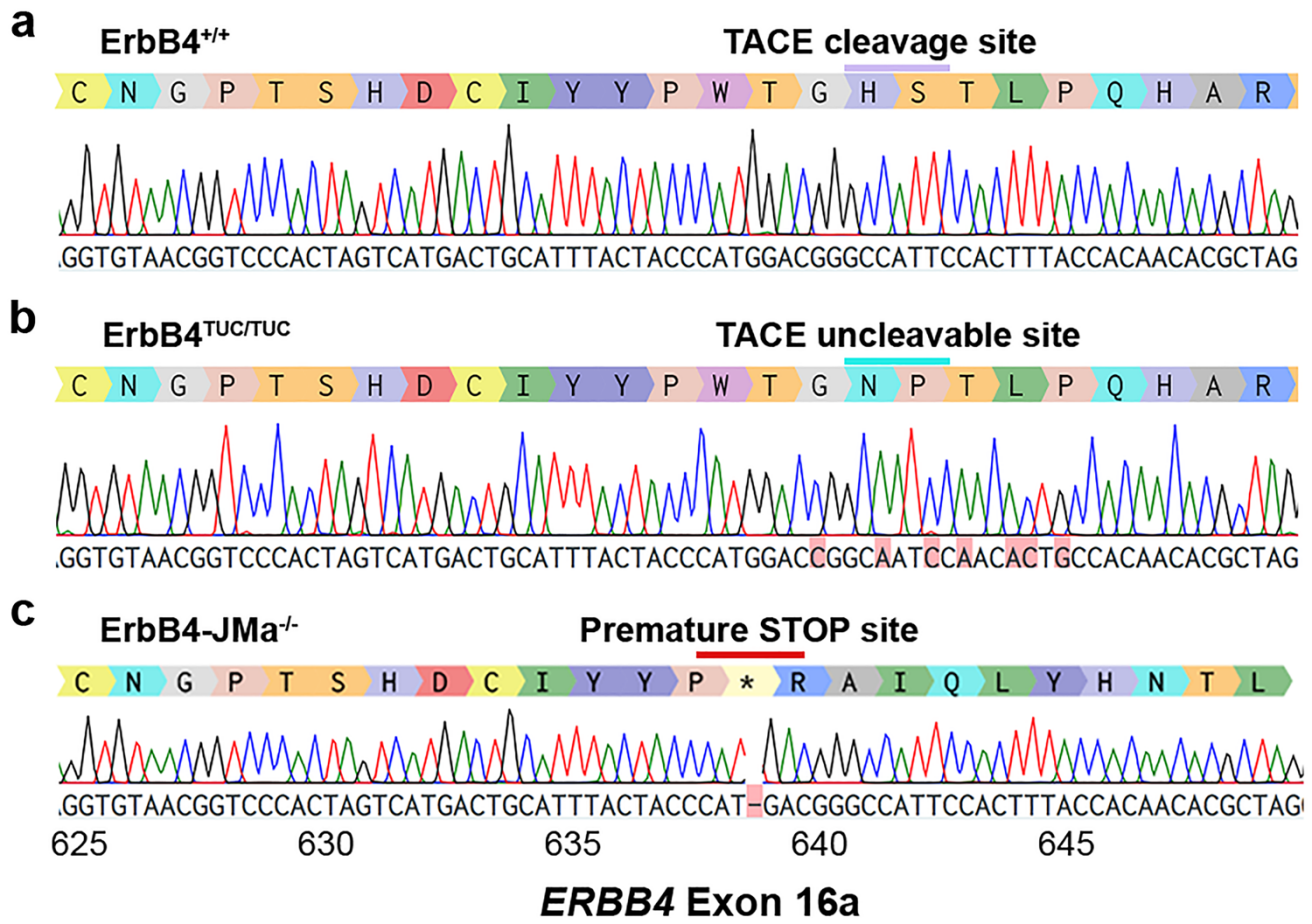
Genomic sequences of the mutations introduced by CRISPR/Cas9 gene editing in the extracellular juxtamembrane domain of ErbB4-JMa. Sanger sequencing of ErbB4 Exon 16 from homozygous ErbB4^+/+^, ErbB4^TUC/TUC^ and ErbB4-JMa^−/−^ mice showing the mutations generated by CRISPR/Cas9 gene editing. (**a**) ErbB4^+/+^ sequence shows the wild type sequence with the histidine-serine cleavage site (purple bar). (**b**) Homology-directed repair resulted in inclusion of the mutated oligonucleotide sequence bearing the H641N;S642P TUC mutations in the ErbB4^TUC/TUC^ mice (teal bar). (**c**) Non-homologous end joining created a premature stop codon due to a single base deletion (red bar), generating ErbB4-JMa^−/−^ mice. This mutation was predicted to result in nonsense-mediated degradation of ErbB4-JMa RNA.

We then used CRISPR/Cas9 gene editing to create mice bearing TUC mutations in *ERBB4* exon 16a to target ErbB4-JMa (Fig. 2a). Briefly, a guide RNA was used to create cut sites in exon 16a to replace the wild type sequences with an ultramer oligonucleotide that introduces the H641N;S642P mutations by homology directed repair. From 39 putative founder mice, genotyping identified two mice with the TUC mutations (H641N;S642P) (Fig. 2b). As can be expected from CRISPR/Cas9 mutagenesis, non-homologous end joining also resulted in two other founders bearing a single base pair deletion that created a premature stop codon within *ERBB4* exon 16a (Fig. 2c). mRNAs containing premature stop codons are usually degraded by nonsense-mediated mRNA decay (NMD) before translation, preventing expression^41^. As the mutation is in *ERBB4* exon 16a, and not 16b, we anticipated ErbB4-JMb expression should not be affected in these mutants, creating an ErbB4-JMa isoform-specific knock out (KO). We named these lines ErbB4^TUC^ for the “TACE uncleavable” mice bearing the mutation H641N;S642P, and ErbB4-JMa^-^ for the mice bearing the premature stop codon. Back-crossing of these mutant to homozygosity showed that, ErbB4^TUC/TUC^ and ErbB4-JMa^−/−^ mice are viable.

**Figure 3:**
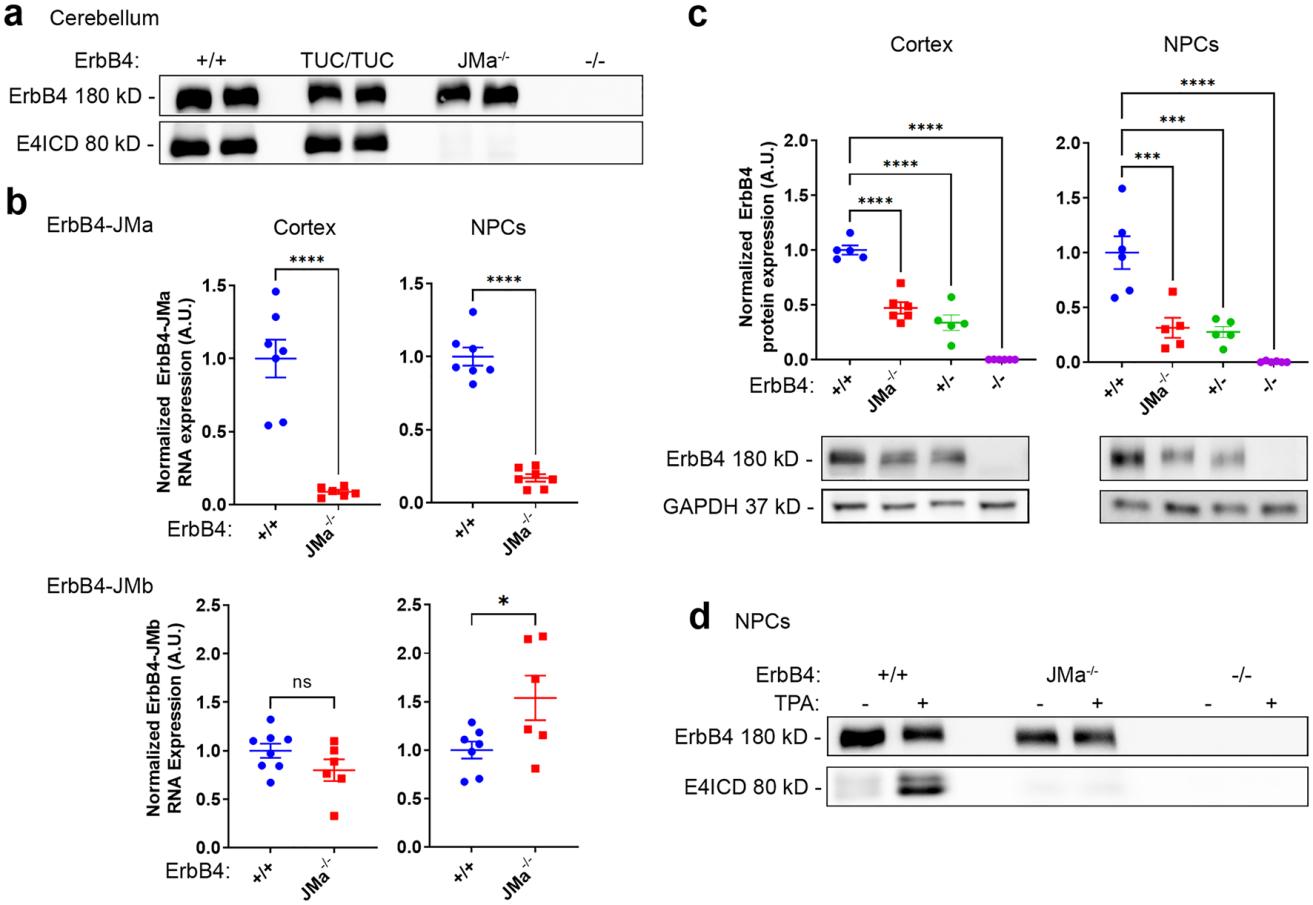
E4ICD formation is present in ErbB4^TUC/TUC^ mice but is abolished in ErbB4-JMa^−/−^ mice due to degradation of ErbB4-JMa RNA. (**a**) ErbB4 immunoprecipitation from cerebellar lysates followed by ErbB4 Western blot shows that the E4ICD band is present in lysates from wild types and ErbB4^TUC/TUC^ tissues but absent in ErbB4-JMa^−/−^ tissues. The image depicting the 80 kD E4ICD band was obtained with a longer exposure than the image depicting the 180 kD full-length ErbB4. (**b**) Isoform-specific qRT-PCR shows that ErbB4-JMa transcripts are almost completely absent from mRNA extracted from NPCs and cortex, whereas ErbB4-JMb RNA expression is relatively normal; n = 6–8 per group. Unpaired t-test was used to evaluate differences. ErbB4-JMa: *p* < 0.0001 for cortex and NPCs, ErbB4-JMb: p = 0.1353 for cortex and p = 0.0400 for NPCs. (**c**) Quantitative Western blot analysis of ErbB4 in NPCs and cortex lysates shows that total ErbB4 levels are decreased in ErbB4-JMa^−/−^ mice to a similar extent as in ErbB4^+/−^ mice; n = 5–6 per group. Ordinary one-way ANOVA followed by Dunnet’s multiple comparisons was used to evaluate differences among the groups to ErbB4^+/+^. Cortex: *p* < 0.0001 for all comparisons. NPCs: ErbB4-JMa^−/−^ *p* = 0.0002; ErbB4^+/−^ *p* = 0.0001; ErbB4^−/−^ *p* < 0.0001. (**d**) TPA treatment induces E4ICD formation in wild type NPCs but not in ErbB4-JMa^−/−^ NPCs. The image depicting the 80 kD E4ICD band was obtained with a longer exposure than the image depicting the 180 kD full-length ErbB4.

To assess the impact of the ErbB4^TUC^ and ErbB4-JMa^-^ mutations on E4ICD generation, we used Western blot (WB) analysis of the adult cerebellum, a part of the nervous system where both ErbB4-JMa and ErbB4-JMb are expressed^8^. Lysates of adult cerebella were subjected to immunoprecipitation with antibodies directed to the carboxy terminal end of ErbB4 and the presence of the full-length receptor (180 kD) and E4ICD (80 kD) were tested by WB. The E4ICD band was clearly detectable in lysates from ErbB4^+/+^ mice and, surprisingly, ErbB4^TUC/TUC^ mice, indicating that the TUC mutations in *ERBB4* exon 16a did not prevent ErbB4-JMa cleavage in vivo (Fig. 3a). However, the ErbB4-JMa^−/−^ lysates lacked E4ICD (Fig. 3a), which most likely is caused by loss of ErbB4-JMa expression. To test this, we performed quantitative RT-PCR (qRT-PCR) on P0 cortex and NPCs, which also express both ErbB4-JMa and ErbB4-JMb (Figs. 3b and S1). As expected, the levels of ErbB4-JMa mRNA were dramatically reduced in ErbB4-JMa^−/−^ tissues, suggesting that it is degraded by NMD, while ErbB4-JMb mRNA expression was relatively normal (slightly increased in NPCs) (Fig. 3b). As could be expected from the loss of ErbB4-JMa mRNA, quantitative WB (qWB) of P0 cortex and NPC lysates showed that the total level of ErbB4 protein was reduced in ErbB4-JMa^−/−^ tissues, being comparable to levels in heterozygous mice (ErbB4^+/−^) (Fig. 3c). The loss of total ErbB4 protein levels in ErbB4-JMa^−/−^ mutants suggested that the levels of canonical signaling could be reduced in tissues derived from these mice, potentially resulting in functional heterozygosity, which could confound interpretation of any phenotype observed in the new KO line. To account for this scenario, in the following experiments we used ErbB4^+/−^ mice as additional controls.

To confirm that the loss of E4ICD in the ErbB4-JMa^−/−^ tissues reflects the loss of ErbB4 cleavage, we used neural precursor cells (NPCs) derived from the E14.5 telencephalon, which express both ErbB4-JMa and ErbB4-JMb^3^ (Fig. S1). Consistent with the findings in cerebellar tissues, TPA treatment induced E4ICD formation in ErbB4^+/+^ NPCs but not ErbB4-JMa^−/−^ NPCs (Fig. 3d). Together, these results show that the ErbB4-JMa^−/−^ mouse is an ErbB4-JMa isoform-specific KO deficient in non-canonical E4ICD signaling, and this mouse line could be used to identify biological processes that require non-canonical ErbB4-JMa signaling in vivo.

### Regulation of GFAP expression by ErbB4 is mediated by ErbB4-JMa non-canonical signaling in vivo

**Figure 4:**
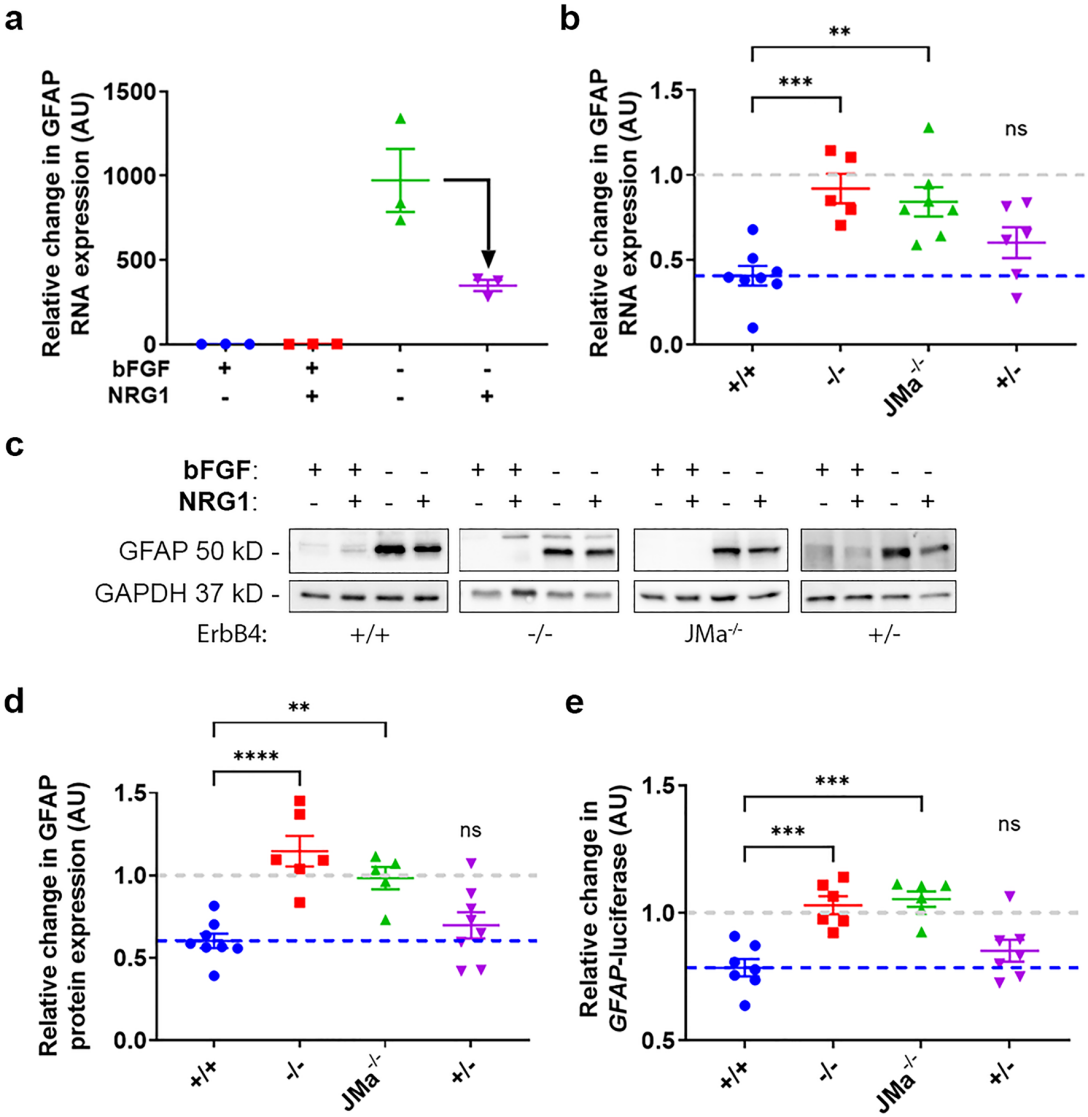
NRG1-induced repression of GFAP expression in NPCs is lost in ErbB4-JMa^−/−^ cells. (**a**) Effects of bFGF removal and/or NRG1 treatment on GFAP mRNA expression in wild type NPCs. NRG1 treatment does not alter GFAP expression in NPCs in medium containing bFGF and NRG1. bFGF removal leads to up to 1000-fold increase in GFAP expression one day later, but addition of NRG1 to the medium at the time of bFGF removal reduces the upregulation by more than half. The graph depicts one experiment with 3 technical repeats, this experiment was performed 8 times with similar results. All points were normalized to the average GFAP expression in NPCs in the presence of bFGF and without NRG1. (**b**) The repressive effect of NRG1 on the upregulation of GFAP mRNA levels after bFGF removal is lost in NPCs from ErbB4^−/−^ and ErbB4-JMa^−/−^ but it is present in ErbB4^+/−^ cells; n = 5–8 per group. Each point represents the ratio of GFAP NGE in NPCs treated with NRG1 vs untreated 24 h after bFGF removal. An ordinary one-way ANOVA followed by Dunnett’s multiple comparisons was used to compare ErbB4^+/+^ NPCs to all other groups. ErbB4^−/−^ *p* = 0.0006; ErbB4-JMa^−/−^ *p* = 0.0011; ErbB4^+/−^ *p* = 0.2110. (**c**) Representative GFAP and GAPDH Western blots of lysates from NPCs for the results shown in panel (**d**). (**d**) The repressive effect of NRG1 on upregulation of GFAP protein levels after bFGF removal in wild type NPCs is lost in NPCs derived from ErbB4^−/−^ and ErbB4-JMa^−/−^ embryos but it is present in ErbB4^+/−^ cells; n = 5–8 per group. GFAP levels were normalized to GAPDH. Each point represents the ratio of normalized GFAP protein levels in NPCs treated with NRG1 vs untreated 24 h after bFGF removal. An ordinary one-way ANOVA followed by Dunnett’s multiple comparison test was used to compare ErbB4^+/+^ NPCs to all other groups. ErbB4^−/−^ *p* < 0.0001; ErbB4-JMa^−/−^ *p* = 0.0046; ErbB4^+/−^ *p* = 0.6350. (**e**) The repressive effect of NRG1 on the activation of a GFAP-promoter luciferase reporter after bFGF removal is lost in NPCs from ErbB4^−/−^ and ErbB4-JMa^−/−^ but it is present in ErbB4^+/−^ cells; n = 6–7 per group. Luciferase activity was normalized using a CMV-Renilla reporter. An ordinary one-way ANOVA followed by Dunnett’s multiple comparison test to ErbB4^+/+^ NPCs was used to compare ErbB4^+/+^ NPCs to all other groups. ErbB4^−/−^ *p* = 0.0003; ErbB4-JMa^−/−^ *p* = 0.0001; ErbB4^+/−^ *p* = 0.4236.

We previously showed that nuclear signaling by E4ICD regulates the timing of astrogenesis by repressing GFAP expression^3^. Specifically, we showed that NRG1-induced E4ICD signaling represses CNTF-induced GFAP expression in rat NPCs. We also showed that mice with complete loss of ErbB4 have increased GFAP expression in the ventricular zone of the developing brain, and that this phenotype can be rescued by expression of constitutively active E4ICD. Therefore, we tested if loss of ErbB4-JMa produces similar phenotypes. We found that GFAP expression in mouse NPCs is strongly induced by removal of bFGF from the culture medium, and that addition of NRG1 reduces the effect of bFGF removal on GFAP expression by more than 50% (Fig. 4a and b). The NRG1-induced repression of GFAP expression after bFGF removal was absent in NPCs derived from both ErbB4-JMa^−/−^ and ErbB4^−/−^ embryos, indicating that ErbB4-JMa is responsible for this effect of NRG1 (Fig. 4b). Importantly, the effect of NRG1 on GFAP expression was normal in ErbB4^+/−^ NPCs (Fig. 4b). As total ErbB4 levels are similar in ErbB4-JMa^−/−^ and ErbB4^−/−^ NPCs (Fig. 3c), this indicates that the loss of the NRG1 response in the ErbB4-JMa^−/−^ cells is not due to the reduction in total full-length ErbB4 expression observed in ErbB4-JMa^−/−^ NPCs. Similarly, NRG1 treatment of NPCs after bFGF removal reduced the levels of GFAP protein (Fig. 4c and d) and repressed the activity of the *GFAP* promoter assessed by a luciferase reporter assay (Fig. 4e and S2) in ErbB4^+/+^ and ErbB4^+/−^ NPCs, but not in ErbB4^−/−^ and ErbB4-JMa^−/−^ NPCs. These results prove that ErbB4-JMa and the direct nuclear signaling by E4ICD repress astrocytic gene expression in vivo as suggested in our previous study^3^. Since ErbB4^+/−^ and ErbB4-JMa^−/−^ have similar levels of full-length ErbB4 and therefore most likely similar levels of canonical signaling, these results indicate that the changes that occur in ErbB4-JMa^−/−^ cells but not in ErbB4^+/−^ counterparts are due to loss of direct nuclear signaling. Furthermore, these results also show that NRG1/ErbB4-JMb signaling is not sufficient to regulate GFAP expression in NPCs.

**Figure 5:**
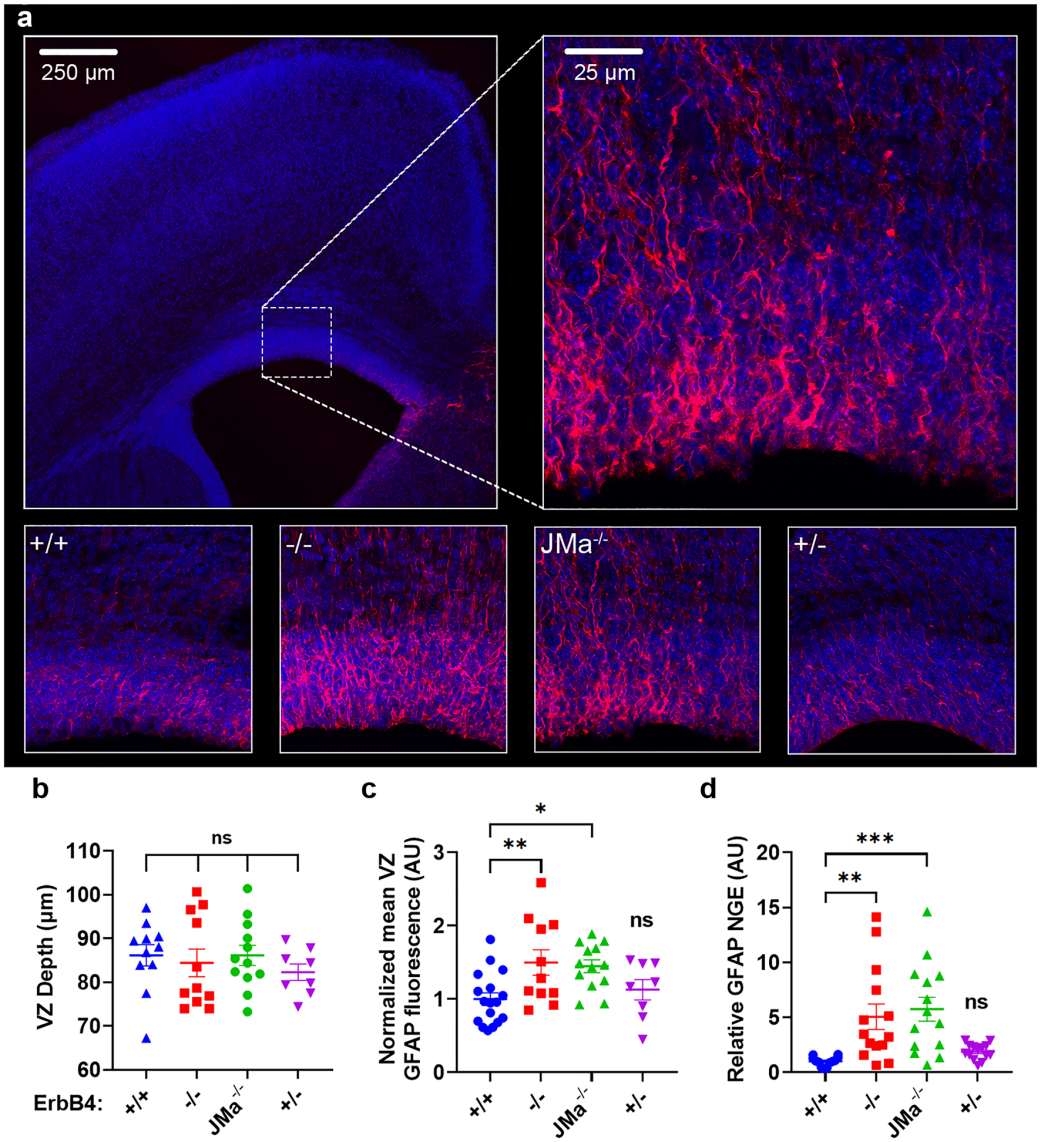
Loss of ErbB4-JMa upregulates GFAP protein expression in the neonatal subventricular zone. (**a**) Top: representative images of the area in the brain neonatal brain in which GFAP expression was analyzed by quantitative immunofluorescence. Bottom: representative images showing staining of sections of ErbB4^+/+^, ErbB4^−/−^, ErbB4-JMa^−/−^, and ErbB4^+/−^ brains. Brightness on all representative images was increased 70% for visibility. (**b**) The depth of the ventricular zone (VZ) is not affected by genotype; n = 8–17 per group. An ordinary one-way ANOVA followed by Dunnett’s multiple comparison test to ErbB4^+/+^ NPCs was carried out to evaluate the differences between the groups. All comparisons were not significant. (**c**) GFAP fluorescence intensity in the VZ is higher in ErbB4^−/−^ and ErbB4-JMa^−/−^ mice compared to ErbB4^+/+^ mice, but not in ErbB4^+/−^ mice; n = 8–12 per group. An ordinary one-way ANOVA followed by Dunnett’s multiple comparison test to ErbB4^+/+^ NPCs was used to compare ErbB4^+/+^ to all other genotypes. ErbB4^−/−^ p = 0.0087; ErbB4-JMa^−/−^ p = 0.0141; ErbB4^+/−^ p = 0.8284. (**d**) GFAP mRNA levels in the cortex at E15.5 are increased in in ErbB4^−/−^ and ErbB4-JMa^−/−^ mice compared to ErbB4^+/+^ mice, but not in ErbB4^+/−^ mice; n = 12–14 per group. An ordinary one-way ANOVA followed by Dunnett’s multiple comparison test to ErbB4^+/+^ NPCs used to compare ErbB4^+/+^ to all other genotypes. ErbB4^−/−^ *p* = 0.0039; ErbB4-JMa^−/−^ *p* = 0.0007, ErbB4^+/−^ *p* = 0.7928.

To determine if loss of ErbB4-JMa alters GFAP expression in vivo, we assessed GFAP levels in the ventricular zone in the neonatal brain using quantitative immuno-fluorescence (Fig. 5a). While ventricular zone (VZ) depth remained the same (Fig. 5b), we found increased GFAP immunofluorescence levels in the VZ of ErbB4^−/−^ and ErbB4-JMa^−/−^ mice compared to ErbB4^+/+^ and ErbB4^+/−^ mice (Fig. 5c), indicating that loss of ErbB4-JMa signaling leads to increased GFAP protein expression in the brain’s germinal layer. Correspondingly, GFAP RNA levels were also elevated in whole the E15.5 developing brain of ErbB4^−/−^ and ErbB4-JMa^−/−^ mice relative to wild types and ErbB4^+/−^ mice (Fig. 5d). Together, these results provide further support to the notion that E4ICD signaling is necessary for the NRG1-mediated regulation of GFAP expression in NPCs in the developing brain. Furthermore, these results validate the ErbB4-JMa^−/−^ mouse model as a tool to explore the roles of ErbB4-JMa and non-canonical ErbB4 nuclear signaling in vivo.

### ErbB4-JMa is not required for heart, mammary gland, and neural ganglia development

**Figure 6:**
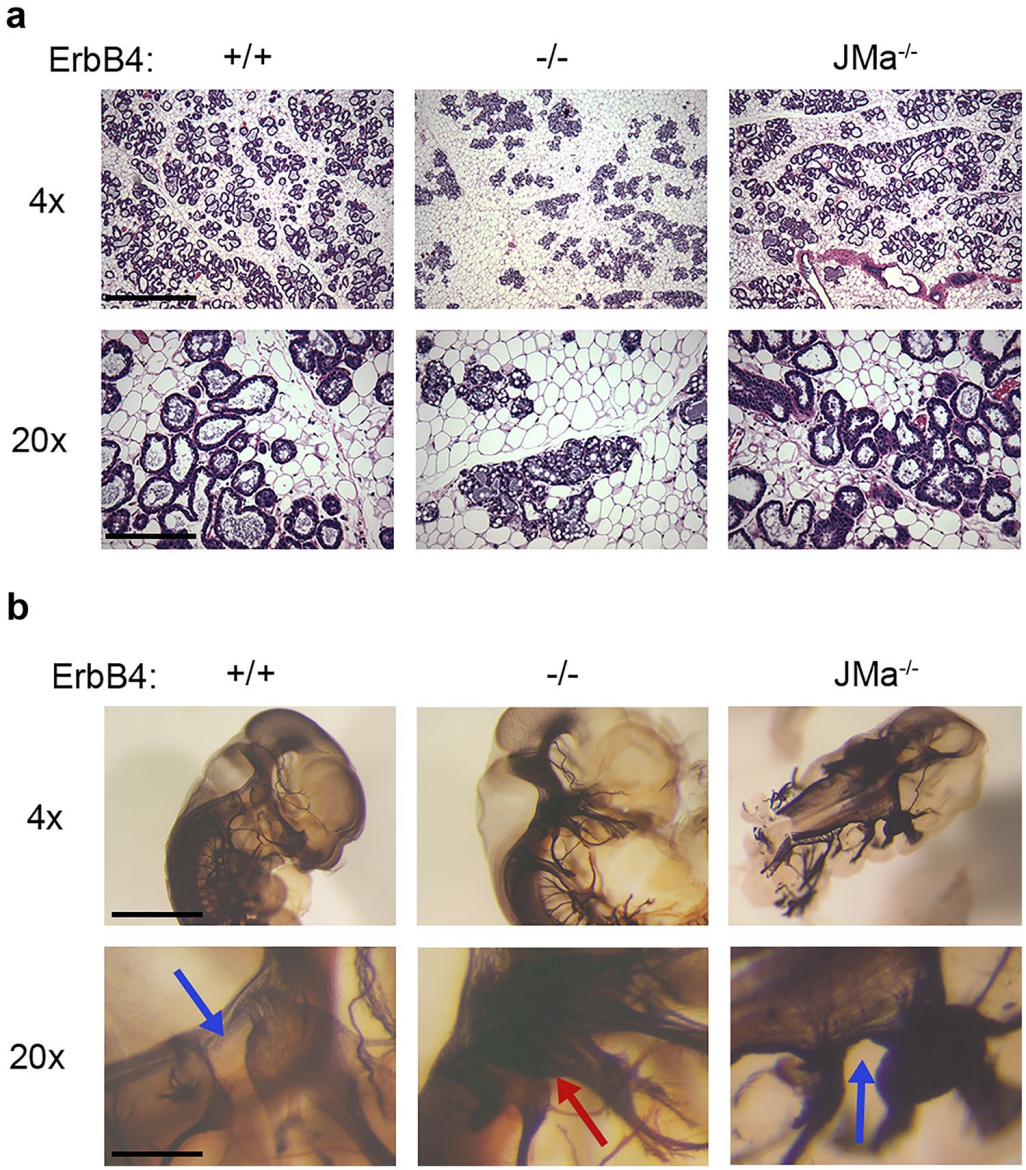
Loss of ErbB4-JMa does not alter mammary gland development and trigeminal ganglion structure. (**a**) Hematoxylin/eosin staining of mammary gland from dams one day after giving birth show that ErbB4^−/−^ mice form fewer ductal structures with vacuolar inclusions in the duct walls than ErbB4^+/+^ dams. ErbB4-JMa^−/−^ dams form regular mammary ducts and wean regular litters. Scale bar represents 50 µm in 4 × and 250 µm in 20 × images. (**b**) Neurofilament immunohistochemistry of E11.5 embryos shows an ectopic axonal bridge between the trigeminal and facial ganglion in ErbB4^−/−^ mice (red arrow) that is not present in ErbB4^+/+^ embryos (blue arrow). This abnormality is absent from ErbB4-JMa^−/−^ embryos (blue arrow). Scale bar represents 50 µm in 4 × and 250 µm in 20 × images.

ErbB4^−/−^ mice have several phenotypes, some of which have been suggested to depend on non-canonical signaling based on in vitro assays^33,35^. Therefore, we tested if these defects are phenocopied by loss of ErbB4-JMa. First, germline ErbB4-KO mice die during early embryonic development due to defects in heart formation^15^. Consequently, studies on ErbB4-KOs have been performed with mice rescued by cardiac-specific expression of ErbB4 under $\alpha$-MHC promoter^16^. Unlike ErbB4^−/−^ mice, ErbB4-JMa^−/−^ mice are viable, and their heart is normal at the gross anatomical level (not shown). Second, ErbB4^−/−^ females have defective lactation due to a deficiency in post-natal mammary alveolar development^16^ and therefore fail to raise their pups, i.e., the early studies of ErbB4^−/−^ mice reported that 82% of pups from ErbB4^−/−^ dams die before weaning^16^. In contrast, the size of litters weaned by ErbB4-JMa^−/−^ dams is not different from those from wild type females (7.87 ± 0.39 vs. 8.47 ± 0.50, respectively; *p* = 0.350, n = 15 litters/genotype). Furthermore, histological analysis of mammary glands from dams one day post-delivery showed that ErbB4^−/−^ mice have abnormal, disorganized development of alveolar epithelial cells with lipid globules within the lumen (Fig. 6a), as previously shown^16^, whereas ErbB4-JMa^−/−^ females develop normal post-delivery mammary alveolar structures (Fig. 6a). Third, ErbB4^−/−^ embryos form an aberrant neuronal bridge between the trigeminal ganglion (gV) and geniculate ganglion (gVII) due to abnormal migration and pathfinding by neural crest cells^21^. This can be visualized by neurofilament immunostaining of whole mount E10.5 embryos^16^, which showed that the abnormal bridge is absent in ErbB4-JMa^−/−^ embryos (Fig. 6b). These results suggest that ErbB4-JMa is not essential for heart, mammary gland and neural ganglia development, but we cannot rule out that ErbB4-JMa^−/−^ mice have subtle defects in these tissues that were not detected with the current level of analysis.

### Identification of the signaling mechanism responsible for ErbB4-mediated gene expression during cortical development using ErbB4-JMa^−/−^ mice

**Figure 7:**
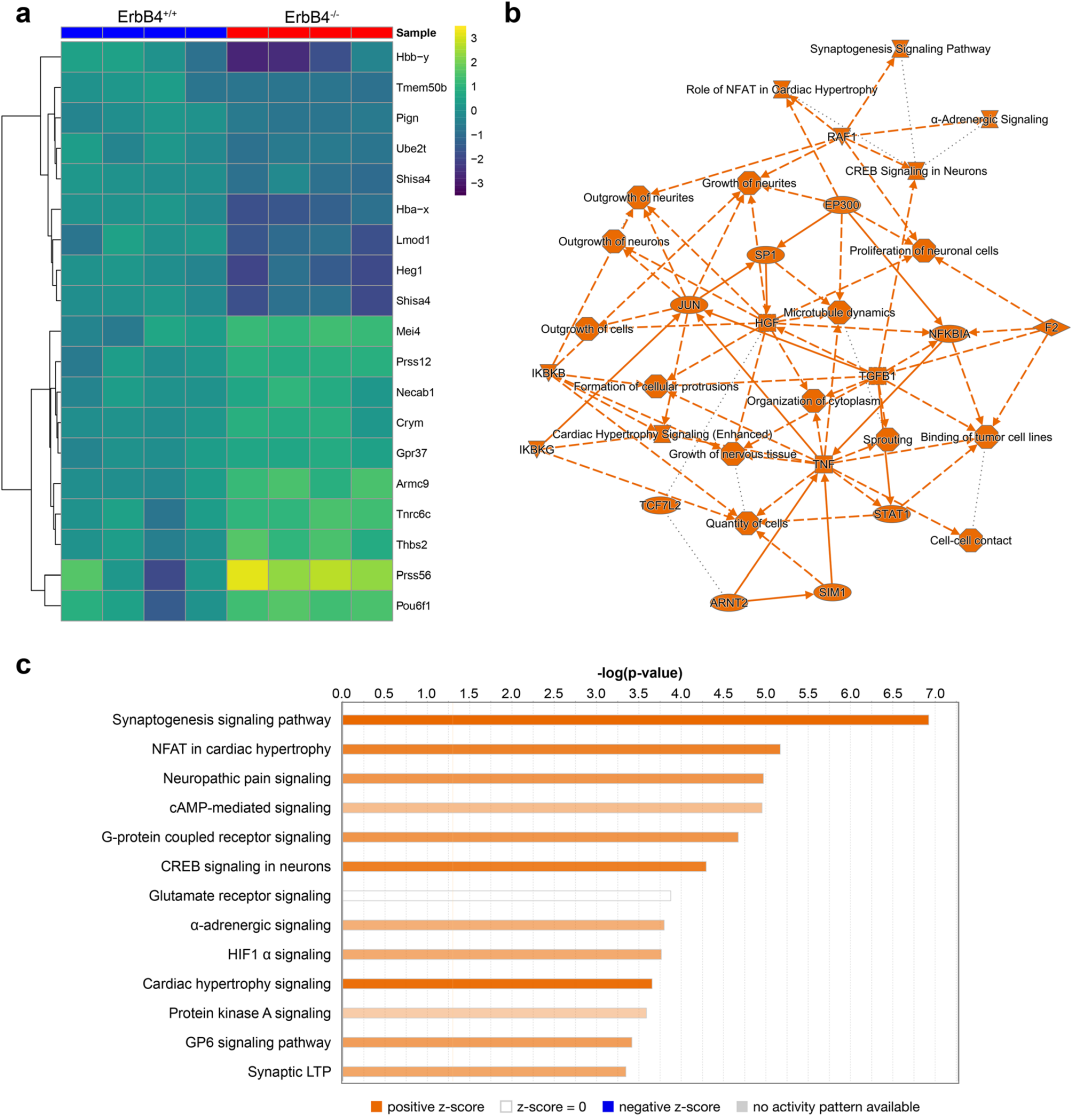
Identification of genes whose expression in the E15.5 cortex is altered by loss of ErbB4. (**a**) Heat map of the differentially expressed genes identified by bulk RNA-sequencing of ErbB4^+/+^ and ErbB4^−/−^ tissues (adjusted *P* value < 0.75 and lfc < 0.58). (**b**) Summary of the Ingenuity Pathway Analysis (IPA) showing hub genes and pathways that are changed by loss of ErbB4. (**c**) Pathways predicted by IPA to be regulated by ErbB4 in the E15.5 cortex; the top 13 pathways are shown ranked by significance.

Having validated that the new mutant line specifically eliminates ErbB4-JMa and thus allows for the distinction between biological processes that depend on ErbB4-JMa or ErbB4-JMb, we set out to identify new genes whose expression is differentially regulated by the two isoforms during cortical development. We first identified genes whose expression is altered by loss of ErbB4 by bulk RNA sequencing (RNA-seq) on ErbB4^+/+^ and ErbB4^−/−^ E15.5 embryonic brain cortex samples. This identified 20 differentially expressed genes (DEGs), 12 upregulated and 8 downregulated in the KO (q value < 0.05, Table 1). Heat map analysis shows that gene expression differences were consistent between genotypes among different replicates (Fig. 7a). Ingenuity Pathway Analysis (IPA) shows that the DEGs are linked to pathways known to be associated with ErbB4, such as outgrowth of cells, neurites and neurons, and growth and proliferation of neurons (Fig. 7b). Pathway analysis of the top 200 upregulated and 200 downregulated genes shows that systems known to be regulated by ErbB4 were altered in ErbB4^−/−^ samples, such as RTK phosphorylation (cAMP, CREB, PKA, GPCRs); synaptogenesis and synaptic plasticity; and breast cancer and tumor microenvironment (Fig. 7c). These analyses validated that the RNA-seq was reliable and consistent with expected roles of ErbB4 in the developing cortex.

**Table 1:**
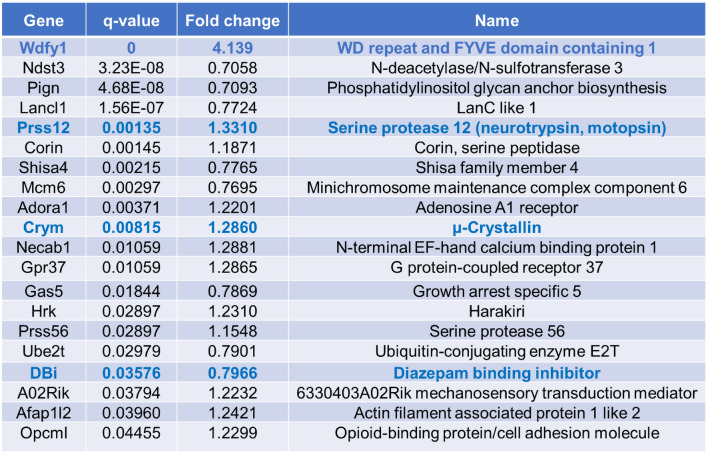
Genes whose expression levels in the E15.5 brain are significantly altered by loss of ErbB4.

Genes analyzed by qRT-PCR are shown in blue.

**Figure 8:**
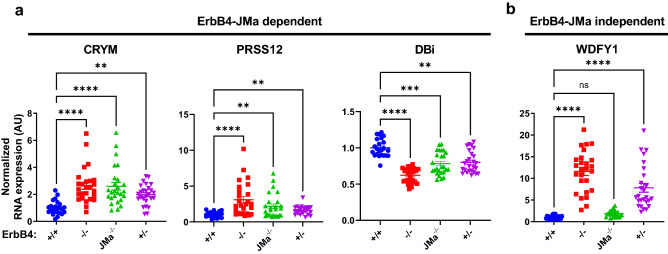
Testing the role of ErbB4-JMa in the regulation of the identified DEGs. (**a**) qRT-PCR analysis shows that expression of CRYM, PRSS12, and DBi expression in the E15.5 cortex is altered in ErbB4-JMa^−/−^, ErbB4^−/−^ and ErbB4^−/+^ mice; n = 11–12 per group. Kruskall Wallis test followed by Dunn’s multiple comparison test to ErbB4^+/+^ was carried out to evaluate the differences between ErbB4^+/+^ and the other genotypes. CRYM: *p* < 0.0001 for ErbB4^−/−^ and ErbB4-JMa^−/−^, *p* = 0.004 for ErbB4^−/+^. PRSS12: *p* < 0.0001 for ErbB4^−/−^, *p* = 0.004 for ErbB4-JMa^−/−^, *p* = 0.0074 for ErbB4^−/+^. DBi: *p* < 0.0001 for ErbB4^−/−^, *p* = 0.004 for ErbB4-JMa^−/−^, *p* = 0.0016 for ErbB4^−/+^. (**b**) In contrast, WDFY is increased in ErbB4^−/−^ and ErbB4^−/+^ animals but not in ErbB4-JMa^−/−^ mice, indicating that their expression does not depend on ErbB4-JMa; n = 11–12 per group. Kruskall Wallis test followed by Dunn’s multiple comparison test to ErbB4^+/+^ was carried out to evaluate the difference between ErbB4^+/+^ and the other genotypes. *p* < 0.0001 for ErbB4^−/−^ and ErbB4^−/+^.

We then used qRT-PCR to determine if some of the DEGs are specifically regulated by ErbB4-JMa, focusing on four genes that have been implicated in the NPCs biology or brain development (blue genes in Table 1). We found that expression of CRYM (µ-crystallin), PRSS12 (serine protease 12), and DBi (diazepam binding inhibitor) is altered in the cortex of both ErbB4-JMa^−/−^ and ErbB4^−/−^embryos (Fig. 8a), showing that the JMa isoform and possibly ErbB4-JMa non-canonical signaling, are involved in their regulation. However, since these mRNA species were also altered in the ErbB4^−/+^ mice, the results do not provide definite proof that these genes are regulated by non-canonical signaling. In contrast, WDFY1 (WD Repeat and FYVE Domain Containing 1) expression is upregulated in the cortex of ErbB4^−/−^ and ErbB4^−/+^ embryos but not in ErbB4-JMa^−/−^ tissues (Fig. 8b), showing that expression of WDFY1 is independent of ErbB4-JMa and most likely is specifically regulated by ErbB4-JMb.

## Discussion

Here, we demonstrate that ErbB4-JMa^−/−^ mice lack E4ICD formation and have defects in the regulation of astrogenesis and GFAP expression as predicted by our prior studies^3,8,9^, validating this mutant line as a novel tool to test the roles of ErbB4-JMa and non-canonical ErbB4 signaling in vivo. Surprisingly, the TUC mutations (H641N;S642P) abolish E4ICD formation in heterologous cells but not in the ErbB4^TUC/TUC^ mouse in vivo. This could reflect differences between ErbB4 glycosylation in vivo and in vitro, since ErbB4-JMa’s preferred TACE cleavage site has been proposed to shift from H641 to P637 upon receptor glycosylation^42^. Alternatively, proteases other than TACE might be able to also cleave ErbB4-JMa on a different site in vivo. Whatever the reason for this result, the different effects of the TUC mutation in vitro and in vivo indicate that, without an animal model eliminating the RTK cleavage, conclusions from studies on cleavage-dependent direct nuclear signal by RTKs based solely on heterologous expression systems should be interpreted with caution.

ErbB4-JMa^−/−^ mice do not phenocopy several characteristics found in mice with complete ErbB4 loss of function, including lethal heart development defects, mammary gland malfunction, and alterations in neural ganglia formation. While we cannot rule out that some subtle phenotypes occur in these tissues in the ErbB4-JMa^−/−^ mice, our results indicate that ErbB4-JMa and its direct nuclear signaling are not essential for these biological processes. This suggests that the in vitro observations that non-canonical signaling by ErbB4 plays a role in cardiac^35^ and mammary gland^43^ development might have been misleading. Along with the results from ErbB4^TUC/TUC^ mice, these findings further emphasize that in vitro observations of non-canonical ErbB4 can be unreliable, and that studies in the intact organism are required to draw conclusions about the roles of non-canonical RTK signaling in general.

Having validated the ErbB4-JMa^−/−^ mouse model by confirming the predicted alterations in GFAP expression, we used it to investigate the role of non-canonical ErbB4 signaling in the regulation of gene expression in the embryonic brain. Of the DEGs identified by bulk RNA-seq on ErbB4^+/+^ and ErbB4^−/−^ embryos, *CRYM, PRSS12* and *DBi* were found to be regulated by ErbB4-JMa. Interestingly, DBi has been shown to influence NPC proliferation through regulation of γ-aminobutyric acid type A receptors (GABAaRs)^44,45^, whose expression is also regulated by ErbB4^46,47^, raising the possibility that these three molecules interact during brain development. The decrease in DBi expression due to loss of ErbB4-JMa may play a role in the early transition from progenitor maintenance to astrogenesis. A more detailed investigation of the interaction between ErbB4-JMa, DBi and GABAaR in the regulation of NPCs may provide further insights into the mechanisms of cortical development. *PRSS12* encodes neurotrypsin, a serine protease, truncation of which causes intellectual disability in humans^48,49^ and mouse models have shown that neurotrypsin modulates hippocampal function and social behavior^50^. Interestingly, expression of *PRSS12* has previously been shown to be regulated by presenilins^51^, raising the possibility that presenilin-dependent ErbB4-JMa signaling might be involved in this process. *CRYM* encodes the protein µ-crystallin, which was initially identified as a thyroid hormone binding protein^52^, and was recently shown to be the most enriched marker of striatal astrocytes in the adult brain^53^, suggesting that ErbB4-JMa may also regulate the expression profile of striatal astrocytes. Further exploration of the interactions between µ-crystallin and ErbB4-JMa could reveal more about their roles in astrogenesis.

In summary, creating the ErbB4-JMa^−/−^ mice has allowed us to expand the understanding of non-canonical ErbB4 signaling in the intact organism. In future studies, the ErbB4-JMa^−/−^ mouse line could be used to identify other developmental and biological processes regulated by ErbB4 and determine if they are mediated by canonical or non-canonical signaling.

## Materials and methods

### Animals

All animals were kept under a 12/12 h light/dark cycle and temperature (21–23 °C) controlled environment and were fed ad libitum with a standard chow diet (5LOD, LabDiet, USA). Animal procedures were reviewed and approved by the University of Michigan Institutional Animal Care and Use Committee, in accordance with the National Research Council Guide for the Care and Use of Laboratory Animals. Mice were housed in an Association for Assessment and Accreditation of Laboratory Animal Care–accredited facility.

All mouse lines were maintained as homozygous, except for ErbB4^−/+^, which were generated from mating of ErbB4^−/−^ males and ErbB4^+/+^ females. ErbB4^−/−^ litters were cross-fostered to ErbB4^+/+^mothers for weaning. The genotypes of mice were confirmed by PCR detection of the transgenes in tail-derived DNA from the ErbB4^+/+^, ErbB4^+/−^, ErbB4^−/−^, ErbB4^TUC/TUC^, ErbB4 JMa^−/−^ and each control mice at weaning and at the end of experiments.

For tissue harvesting, experiments were performed in 2–3 month-old mice. For embryonic harvesting, breeding cages of homozygous mouse lines (or ErbB4^−/−^ males with ErbB4^+/+^ females for ErbB4^−/+^ embryos) were established, and confirmation of vaginal plugging was used for embryonic dating. Positive plug date has been denoted as embryonic day 0.5 (E0.5). Following vaginal plugging, females were euthanized at the indicated time points for embryonic tissue collection.

### CRISPR/Cas9 gene editing to create mutant mice

Mouse lines were generated in collaboration with the Transgenic Animal Model Core, University of Michigan. Two single guide RNAs (sgRNA) (sequences: AAGTGGAATGGCCCGTCCAT and CGTGTTGTGGTAAAGTGGAA) were designed to create cut sites in the ErbB4-JMa sequence within Exon 16. These guides were expected to create a double strand break and introduce the ultramer oligonucleotide bearing the H641N;S642P mutations by homology directed repair, replacing the wild type sequence with the mutant sequence in exon 16. Alternatively, repair of the chromosome break by non-homologous end joining was expected to create other mutations in ErbB4 exon 16. The sgRNAs were co-injected into fertilized C57BL/6 eggs with the CRISPR/Cas9 components (px3330 plasmid, Addgene) and the ultramer oligonucleotide bearing the mutations. From more than 300 injections with both sgRNAs we received 39 putative founder mice. Sequencing analysis of ErbB4 Exon 16 identified 3 mice with the correct mutations (H641N;S642P) and 20 with other mutations. All founders with discernable H641N;S642P mutations and 12 other founders were crossed with wild type mice to create obligatory heterozygotes and ErbB4 Exon 16 sequence of the progeny was analyzed. Of the founders, one mouse had only the H641N;S642P mutation and 2 had progeny carrying a single base pair deletion that creates a premature termination codon within *ERBB4* exon 16. Male and female heterozygote offspring from the founders were inbred to create homozygotes and back-crossed onto the C57Bl6 background. ErbB4-TUC (for the “TACE uncleavable” mice bearing the mutation H641N;S642P) and ErbB4-JMa^-^ (for the mice bearing a premature termination codon which will result in NMD of ErbB4-JMa transcripts) were the names given to the alleles.

### Genotyping mutant mice

To determine mutations in *ERBB4* exon 16 of founder mice Sanger sequencing was carried out on PCR product of *ERBB4* exon 16. PCR amplification of crude genomic lysates from ear biopsies was done using the following primers: Forward: AGA ATG TGG CGC ATC CAG TA; and Reverse: TGC TCT CAT AAT TCC AAT ATG TGC T.

To genotype ErbB4-JMa^−/−^ mice, the primers above were used to amplify exon 16 by PCR. The PCR product was then subjected to restriction enzyme digest with NcoI to create discernible products.

To genotype ErbB4^TUC/TUC^ mice, two PCRs were carried out on crude genomic lysates using forward primers that are specific to the wild type sequence or the TUC mutations as follows: WT Forward Primer: GAC GGG CCA TTC CAC TTT A; TUC Forward Primer: ACC GGC AAT CCA ACA CTG C; and Universal Reverse Primer: TGC TCT CAT AAT TCC AAT ATG TGC TTT AAT C.

ErbB4^+/+^, ErbB4^−/+^ and ErbB4^−/+^ mice were genotyped as described previously^3,16^.

### Cell culture and transfections

Human embryonic kidney (293 (HEK-293) CRL-1573, ATCC) cells or mouse neuro 2A (N2A) cells were maintained in DMEM GlutaMAX (Gibco) with 10% fetal bovine serum (Atlanta) and 10 units/mL penicillin and 0.1 mg/mL streptomycin in a humidified 5% CO2/95% air incubator at 37 °C. All transfections were carried out with Lipofectamine 3000 (Invitrogen) according to the manufacturer’s protocol.

### Site-directed mutagenesis

Site-directed mutagenesis was carried out on the ErbB4-JMa-CYT2 plasmid in pcDNA3.1 using the QuikChange Site-Directed Mutagenesis Kit (Aligent) according to the guidelines. The following primers were used to create H641N;S642P: Forward: TAC TAC CCA ATG GAC GGC AAT CCC ACT TTA CCA CA; and Reverse: TGT GGT AAA GTG GGA TTG CCG TCC ATT GGG TAG TA.

### Neural precursor cell cultures

Timed pregnant females were euthanized via cervical dislocation at E14.5. Embryos were dissected in ice-cold phosphate-buffered saline (PBS) under sterile conditions, and the whole telencephalon without meninges was collected in a 15 mL conical tube containing PBS on ice. PBS was removed and cortices were dissociated into single cells with StemPro Accutase (ThermoFisher) for 5 min at RT, triturated then centrifuged at 2,000 rpm for 2 min. Next, the pellets were resuspended in NPC media (DMEM GlutaMAX (Gibco) with 2% B27 without vitamin A (Invitrogen), 10 units/mL penicillin and 0.1 mg/mL streptomycin). NPCs were seeded as neurospheres in T75 flasks at 500,000 cells/mL and expanded for 2 days in NPC media supplemented with epidermal growth factor (EGF, 20 ng/mL) and basic fibroblast growth factor (bFGF, 20 ng/mL) in a humidified 5% CO2/95% air incubator at 37 °C. Growth factors and B27 were replenished daily. On day 3, neurospheres were dissociated with StemPro Accutase (ThermoFisher) into a single cell suspension and reseeded for NPC expansion or plated in NPC media supplemented with bFGF (20 ng/mL) onto plates coated with Poly-L-Lysine (Sigma) and Fibronectin (Corning).

To assess GFAP protein and mRNA levels, NPCs were plated at 500,000 cells per well in 12-well plate format. Half of the media was replaced after one day, replenishing bFGF, then on the second day all the media was replaced with or without bFGF (to induce astrocytic differentiation and GFAP expression) and treated with 2 nM NRG1 (R&D Systems) or vehicle. After 24 h, cells were lysed in 100 µl RIPA buffer (Sigma) on ice to assess protein levels by western blotting or total RNA was extracted according to RNeasy Mini Kit (Qiagen) for qRT-PCR.

### Luciferase assay

To assess GFAP promoter activity by luciferase assay, NPCs were plated at 800,000 cells per well in 24-well plate format. Half of the media was replaced after one day, replenishing bFGF and cells were co-transfected with a *GFAP*-promoter luciferase construct and *CMV-*Renilla^3^ at a 50:1 ratio using Lipofectamine 3000 (Invitrogen). After 24 h the media was replaced with or without bFGF and treated with 2 nM NRG1 (R&D Systems) or vehicle for 2 days with bFGF and NRG1 replenishment after 1 day. GFAP-promoter driven luciferase activity was measured using the Dual-Luciferase Assay Kit (Promega), following the manufacturer’s instructions on a BioTek plate reader. Relative values were obtained by normalizing firefly luciferase to Renilla luciferase in technical triplicates. The ratio of GFAP-luciferase with and without NRG1 treatment (without bFGF) is displayed for each biological replicate of each genotype.

### Western blotting

Cells were treated as described and lysed with ice cold RIPA buffer (Sigma-Aldrich) or triton-based lysis buffer (Cell Signaling Technologies) with protease and phosphatase inhibitors (Invitrogen) for 10 min. Cerebellum tissue was incubated at 37 °C for 45 min prior to dounce homogenization in RIPA buffer. Lysates were vortexed and centrifuged at 14,000 rpm for 10 min and supernatant protein concentration was standardized via BCA assay (Pierce) and diluted in 4 × Laemmli buffer (Bio-Rad) with 10% β-mercaptoethanol and run on 6% SDS polyacrylamide gels or 7.5% or 10% Mini-PROTEAN TGX Stain-Free Precast Gels (BioRad). Protein was transferred from the gel onto 0.45 µm pore PVDF membrane for 1.5 h at 15 V with a semi-dry transfer unit. Membranes were then incubated in 5% bovine serum albumin (Sigma-Aldrich) in 0.2% Tween-20 in TBS (TBS-T) blocking solution for 1 h and then primary antibody (1:1000, ErbB4, phospho-ErbB4, Erk, phospho-ERK, Cell Signaling Technologies; 1:1000 GFAP, Dako; 1:5000 GAPDH, Abcam) in blocking solution overnight at 4 °C. The next day, blots were washed in TBS-T, then incubated in HRP-conjugated secondary antibody (1:1000 goat anti-rabbit or 1:1000 goat anti-mouse, Cell Signaling Technology) in blocking solution for 45 min. All blots were exposed with SuperSignal™ West Femto Maximum Sensitivity Substrate (Thermo Scientific) and imaged on a Bio-Rad Chemidoc and analyzed using Bio-Rad Image Lab software. To quantify the effect of NRG1 on GFAP protein expression after removal of bFGF, GFAP signal intensity was normalized to GAPDH intensity for each sample and the ratio of GFAP expression with and without NRG1 treatment was calculated.

### RNA extraction and quantitative RT-PCR

Following RNA extraction with Qiagen RNeasy Kit with on-column DNase digestion (Qiagen 74004), RNA concentration and quality was assessed on Bio Tek plate reader. 1 µg of RNA per sample was transcribed into cDNA using an iScript cDNA Synthesis Kit (Bio-Rad) following manufacturer’s instructions and diluted 1:4 in nuclease free water. Quantitative PCR was performed using iTaq SYBR Green Supermix (Bio-Rad) on a Bio-Rad CFX96 Thermocycler in 96 well format or an Applied Biosystems QuantStudio 5 Real-Time PCR System in 384 well format. Each well contained 5 μL iTaq SYBR Green Supermix, 3 pM of each forward and reverse primers and 2.5 μL diluted cDNA. The thermal cycler was run as follows as follows: 95 °C for 30 s followed by 39 cycles of 95 °C for 5 s and 60 °C for 30 s. Normalized Gene Expression (NGE) was calculated using the efficiency of each primer with the following formula: [efficiency_target^–CT^_target_/efficiency_reference^–CT^_ref_]. The primers used for qRT-PCR are listed in Table 2. To quantify GFAP mRNA levels, NGEs were calculated using RPL19 expression as a reference. To determine the effects of NRG1 on GFAP mRNA levels after removal of bFGF, GFAP the ratio of GFAP NGE with and without NRG1 treatment was calculated.

**Table 2:** List of primers used for qRT-PCR.

Gene	Forward primer	Reverse primer
ErbB4-JMa	GAAATG TCC AGA TGG CCT ACA GGG	AAT GCA GTC ATG ACT AGT GGG ACC
ErbB4-JMb	GCA TCG GCC TGA CGG ATA GA	GTC AAA GCC ATG ATC ACC AGG A
GFAP	GCA GGA GTA CCA GGA TCT ACT	TGG AGG TTG GAG AAA GTC TGT
WDFY1	ACC ATC CGA GTA TGG CTG AAA	CCT GCT GTC GTG GTG GTA TG
PRSS12	GGC AGA CCT TGG TGC TTC TAT	CCA CCA ACA AGG CGA ATG AC
CRYM	GTC CAG GCG TAC AGT CAC TA	AGC CTC CTG CAC TGA TGA AC
DBi	GTG GAAAAG GTA GAC GAG CTA AAG A	TAC AGA GGG AGG AGG AGC AGA G
RPL19	ACC TGG ATG AGAAGG ATG AG	ACC TTC AGG TAC AGG CTG TG

### Immunoprecipitation

Cerebellum samples were obtained by dissection of adult mouse brain and stored in ice cold DMEM. Once all samples were collected, they were incubated at 37 °C for 45 min, washed on PBS and lysed in ice cold 500ul RIPA buffer (Invitrogen) using a dounce homogenizer, vortexed and then centrifuged for 10 min at 14,000 rpm and the supernatant was collected.

NPCs were plated in 10 cm dishes at 4 million cells per dish, with two dishes per condition in bFGF-containing NPC media. After 2 days of growth, replenishing half the media and bFGF each day, cells were treated with 100 ng/mL TPA (12-O-Tetradecanoylphorbol-13-Acetate, Cell Signaling Technologies) for 45 min, washed with ice cold PBS and lysed with 500ul lysis buffer (Cell Signaling Technologies). Lysates were subjected to 3 × 5 s sonication using a probe sonicator, then centrifuged at 14,000 rpm for 10 min and supernatants were collected.

An ErbB4 WB was carried out on the supernatant to normalize input levels of total ErbB4 using 40 µl of the sample, while the remaining lysate was frozen at − 80 °C. Lysates were defrosted on ice and diluted to equal total ErbB4 levels in RIPA buffer (ErbB4^−/−^ samples were diluted to the same protein level as the highest concentration sample). ErbB4-conjugated agarose beads (Santa Cruz) were washed in RIPA buffer and 30 μl beads were added to 500 μl of each sample and incubated at 4 °C overnight with rotation. Beads were washed four times in RIPA buffer and the sample was eluted in 1.5 × Laemmli sample buffer (Bio-Rad) for 5 min at 95 °C. Western blot was carried out with ErbB4 antibody (1:1000, ErbB4, Cell Signaling Technologies). Samples from input, IP and supernatant were analyzed by anti-ErbB4 Western blot to measure the levels of the 80 kD E4ICD and full length 180 kD ErbB4.

### P0 Cortex immunofluorescence

Whole brains were dissected from P0 pups in the afternoon following birth and fixed in 4% PFA at 4 °C overnight and then cryopreserved in 30% sucrose in PBS for 2 days at 4 °C. Brains were embedded in OCT Compound (Fisher) and snap frozen in isopentane on dry ice. Frozen brains were cryosectioned at 50 μm into antifreeze solution and stored at −20 °C. Sections were washed/permeabilized in TBS 0.2% Triton (TBS-T) for 20 min and blocked in 5% normal goat serum in TBS-T for 1 h. Sections were then incubated overnight at 4 °C in primary antibody (1:1000. GFAP, Dako) then washed in TBS-T at room temperature. Sections were incubated in secondary antibody (1:1000, Alexa fluor 564 nm goat anti-rabbit, Invitrogen) for 1 h at room temperature then washed in TBS-T and coverslipped with Fluoro-Gel II with DAPI (Electron Microscopy Services) on Superfrost plus slides (Fisher). For analysis, confocal images were taken at a magnification of 63 × at the same location in the ventricular zone with a 12 μm and a 2 μm interval z-step, by a blinded investigator. The average GFAP intensity per ventricular zone area was calculated using Fiji software. Biological replicates were the average of the left and right side of the cortex at the position indicated in the figure.

### Neurofilament immunochemistry

Embryos were taken at E11.5 directly into 4% PFA and incubated overnight at 4 °C, then kept at 4 °C in 0.4% PFA. Embryos were washed in TNT, then dehydrated in a methanol series and incubated in Dents solution (3% hydrogen peroxide, 70% methanol, and 20% DMSO) overnight. Embryos were incubated for 2 days TNT and for 2 days in 5% normal horse serum in TNT (HS-TNT) block, then for 5 days in 0.75 ug/mL 2H3 antibody (Developmental Studies Hybridoma Bank) in HS-TNT. Embryos were washed in TNT, then were incubated for 3 days in 1:250 horse anti-mouse IgG HRP-conjugated Ab (Vector Labs) in HS-TNT then washed in TNT. Signal was developed according to Vectastain DAB Kit (Vector Labs) then washed in TNT for 45 min five times and dehydrated in a methanol grade. Tissue was cleared in BABB solution (1:2 benzyl alcohol/benzyl benzoate) and stored in BABB in glass containers. Images were taken on a dissecting microscope with camera at the magnifications indicated.

### Mammary gland histology

At post-natal day 1 (P1) the number 4 inguinal mammary gland was dissected from the female mice. Mammary glands were spread onto Superfrost plus slides and fixed in 4% PFA overnight. Then, mammary glands were paraffin embedded and processed for haemotoxylin and eosin staining and imaged at 4 × and 40 × magnification using bright field microscopy.

### Statistical analysis

All statistical analyses were performed using GraphPad Prism 9.3.1, except for RNA-seq analysis, described below. Normality was assessed using a Shapiro-Wilks test. For data with two groups, an unpaired t-test was used. For data with multiple groups, an ordinary one-way ANOVA with Dunnett’s multiple comparisons to control ErbB4^+/+^ data was used for parametric statistical analysis. Kruskall Wallis followed by Dunn’s multiple comparisons to control ErbB4^+/+^ data was used for non-parametric statistical analysis. Bars for all graphs represent mean ± SEM.

### RNA-sequencing and analysis

Pregnant females were euthanized via cervical dislocation. E15.5 embryos were placed in ice-cold PBS, brains were dissected, and the cortical hemispheres were isolated. The meninges and ganglionic eminences were removed from the cortex, and the tissue was stored in RNAlater (Invitrogen) until RNA extraction. RNA was extracted from wild type (WT) and ErbB4 KO dorsal cortex using the Qiagen RNeasy Mini Kit with on-column DNase digestion (Qiagen), then analyzed with a BioAnalyzer to measure RNA quality. RNA with RNA integrity numbers (RINs) greater than 8 were sequenced (n = 4 of each genotype). Non-strand specific polyA-selected cDNA libraries were prepared. Single-end sequencing was then completed with read lengths of 50 nucleotides using an Illumina HiSeq-4000 Sequencing System. cDNA library preparation and sequencing were carried out by the University of Michigan DNA Sequencing Core. Sequences were mapped to the mouse genome (mm9) using HISAT, transcript counts obtained with HTseq-count, and differential gene expression analysis completed using DESeq2 with Galaxy software. *p* values adjusted for multiple comparisons (q-value) < 0.05 indicated genes with statistically significant differences. Differential expression data was submitted to Qiagen’s Ingenuity Pathway Analysis (IPA) software-version 70750971 (Qiagen Inc., https://digitalinsights.qiagen.com/IPA) using core analysis of up- and down-regulated expressed genes. Top-scoring enriched pathways, functions, upstream regulators, and networks for these genes were identified utilizing the algorithms developed for Qiagen IPA software based on Qiagen’s IPA database of differentially expressed genes^54^.

The datasets generated and analyzed during the current study have been submitted to NCBI Gene Expression Omnibus (GSE202063).

## Supplementary Information

Supplementary Information.

## Data Availability

All RNA-seq datasets are publicly available at Gene Expression Omnibus under accession number GSE202063.
