# Author Correction: Identification of in vivo roles of ErbB4-JMa and its direct nuclear signaling using a novel isoform-specific knock out mouse

**DOI:** 10.1038/s41598-022-25021-8

**Published:** 2022-12-01

**Authors:** Robert Doherty, Brenna L. MacLeod, Megan M. Nelson, Mostafa M. H. Ibrahim, Beatriz C. Borges, Nada W. Jaradat, Matthew C. Finneran, Roman J. Giger, Gabriel Corfas

**Affiliations:** 1 Department of Otolaryngology-Head and Neck Surgery, Kresge Hearing Research Institute, University of Michigan Medical School, Medical Sciences I Building, Rm. 5428, 1150 West Medical Center Drive, Ann Arbor, MI 48109-5616 USA; 2 Neuroscience Graduate Program, University of Michigan Medical School, Ann Arbor, MI 48109 USA; 3 Department of Cell and Developmental Biology, University of Michigan Medical School, Ann Arbor, MI 48109 USA

Correction to: *Scientific Reports* https://doi.org/10.1038/s41598-022-21598-2, published online 14 October 2022

The original version of this Article contained an error in the Abstract.

“Furthermore, we identify genes whose expression during cortical development is regulated by ErbB4, and show that the expression of three of them, *CRYM* and *DBi*, depend on ErbB4-JMa whereas *WDFY1* relies on ErbB4-JMb.”

now reads:

“Furthermore, we identify genes whose expression during cortical development is regulated by ErbB4, and show that the expression of three of them, *CRYM*, *PRSS12* and *DBi*, depend on ErbB4-JMa whereas *WDFY1* relies on ErbB4-JMb.”

The original Article has been corrected.

