# Supplementary material for: Identification of in vivo roles of ErbB4-JMa and its direct nuclear signaling using a novel isoform-specific knock out mouse
Source: Sci Rep. 2022 Oct 14;12:17267. doi: 10.1038/s41598-022-21598-2 (PMC9568506; doi:10.1038/s41598-022-21598-2)
Supplement: Supplementary file 1 — Supplementary Information. [file 41598_2022_21598_MOESM1_ESM.docx]

**Supplementary Information**

**Fig. S1. Relative levels of ErbB4-JMa and JMb in the cortex and NPCs.** ErbB4 isoform-specific qPCR from ErbB4^+/+^ samples shows the relative levels of ErbB4-JMa and ErbB4-JMb in NPCs, E15.5 and P0 cortex. NPCs express more ErbB4-JMa, whereas in both E15.5 and P0 cortex, there is higher ErbB4-JMb expression. Unpaired t-test was used for statistical analysis. NPCs: p=0.0001; E15 Cortex: p=0.0416; P0 Cortex: p<0.0001.

**Fig. S2. Effects of bFGF removal and/or NRG1 treatment on GFAP-luciferase reporter activity in wild type NPCs.** NRG1 treatment does not alter GFP expression in NPCs in medium containing bFGF and NRG1. bFGF removal leads to up to three-fold increase in GFAP expression one day later, but addition of NRG1 to the medium at the time of bFGF removal reduces the upregulation. Luciferase activity was normalized to *CMV-Renilla* activity. The graph depicts one experiment with 3 technical repeats, this experiment was performed 8 times with similar results.


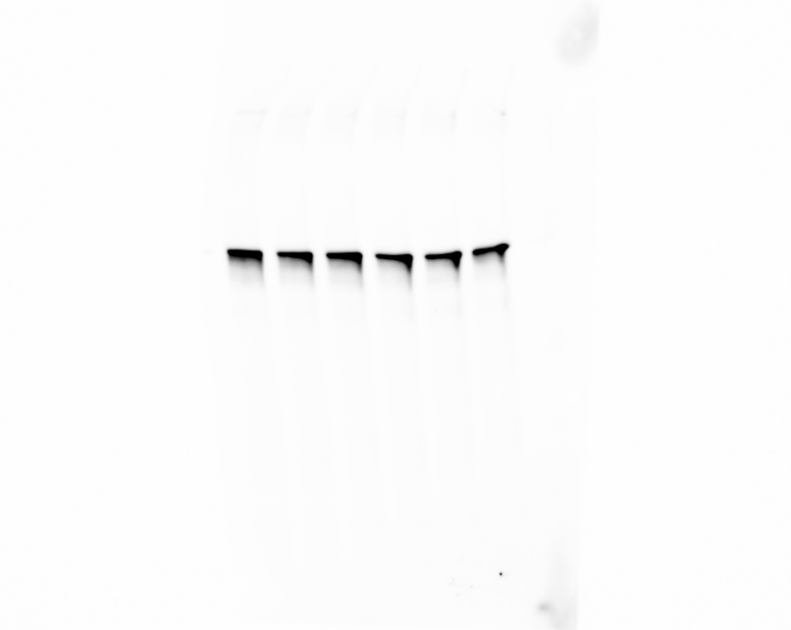


JMb JMa TUC

- + - + - +

**Fig. S3.** Full image of ErbB4 Western blot on lysates of transfected N2A cells for Fig 1b. Square shows area cropped for figure.

JMb JMa TUC

- + - + - +


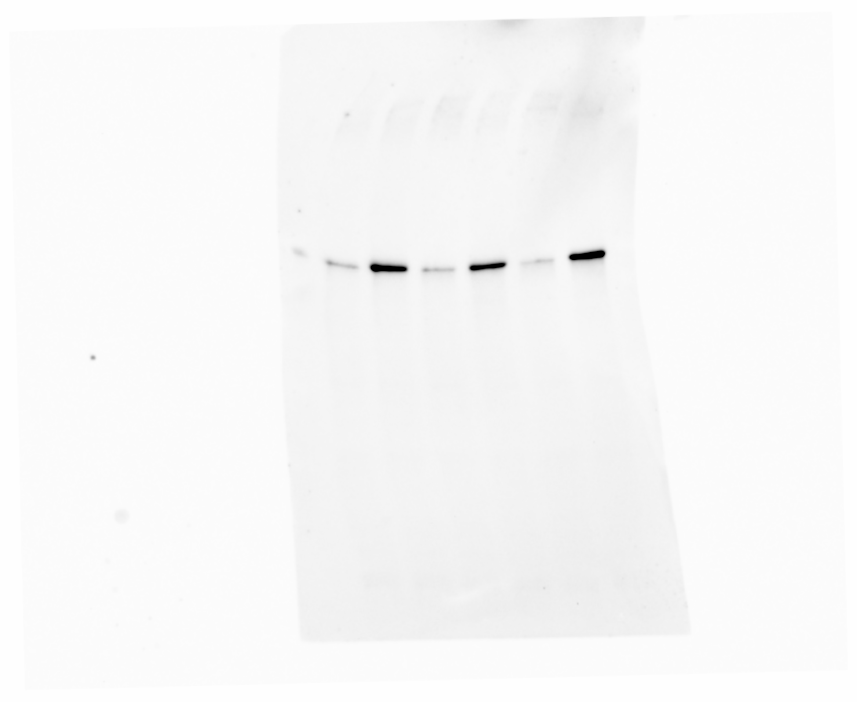


**Fig. S4.** Full image of phospho-ErbB4 WB on lysates of transfected N2A cells for Fig 1b. Square shows area cropped for figure.


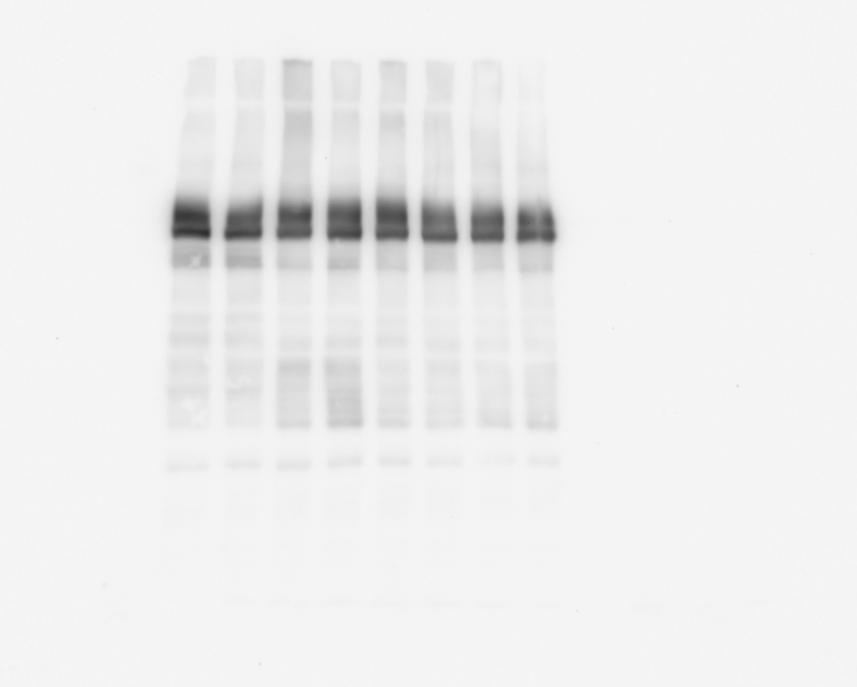


JMb JMa TUC

- + - + - +


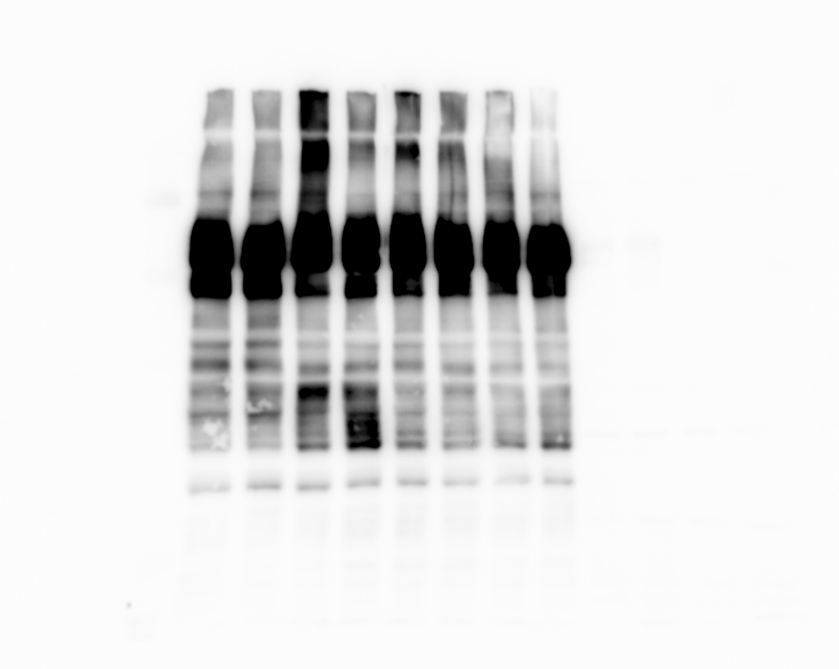


JMb JMa TUC

- + - + - +

**Fig. S5.** Full images of ErbB4 Western blot on lysates of transfected HEK-293 cells to visualize for 180 kD ErbB4 band (top) and longer exposure to visualize for 80kd E4ICD band (bottom) for Fig 1c. Square shows area cropped for figure.


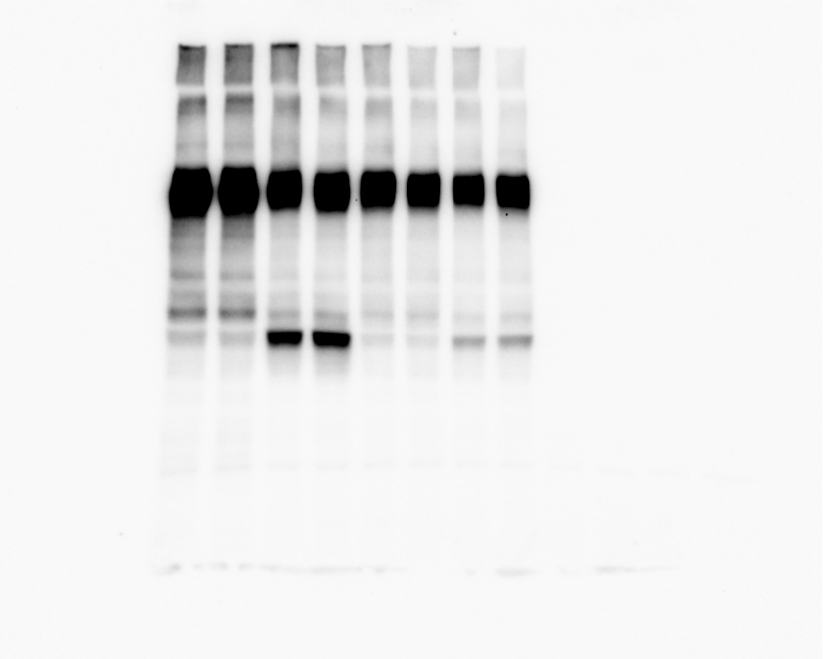


JMb JMa TUC

- + - + - +

**Fig. S6.** Full image of phospho-ErbB4 Western blot on lysates of transfected HEK-293 cells to visualize the 80kd E4ICD band for Fig 1c. Square shows area cropped for figure.

JMb JMa TUC

- + - + - +


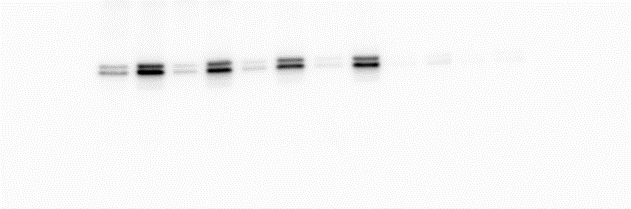


**Fig. S7.** Full image of phospho-ERK Western blot for Fig 1c. The membrane was cut to include relevant molecular weights. Square shows area cropped for figure.


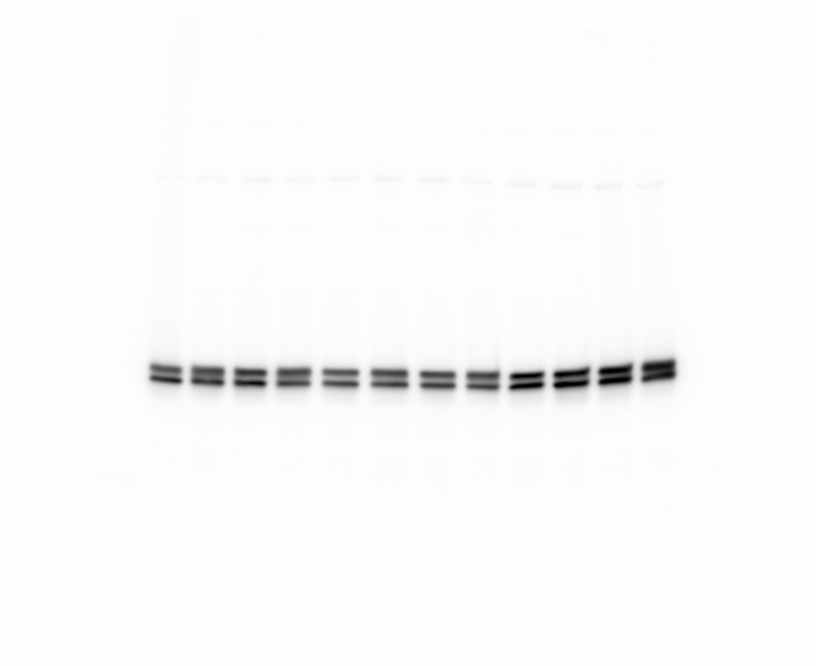


JMb JMa TUC

- + - + - +

**Fig. S8.** Full image of ERK Western blot for Fig 1c. The membrane was cut to include relevant molecular weights. Square shows area cropped for figure.


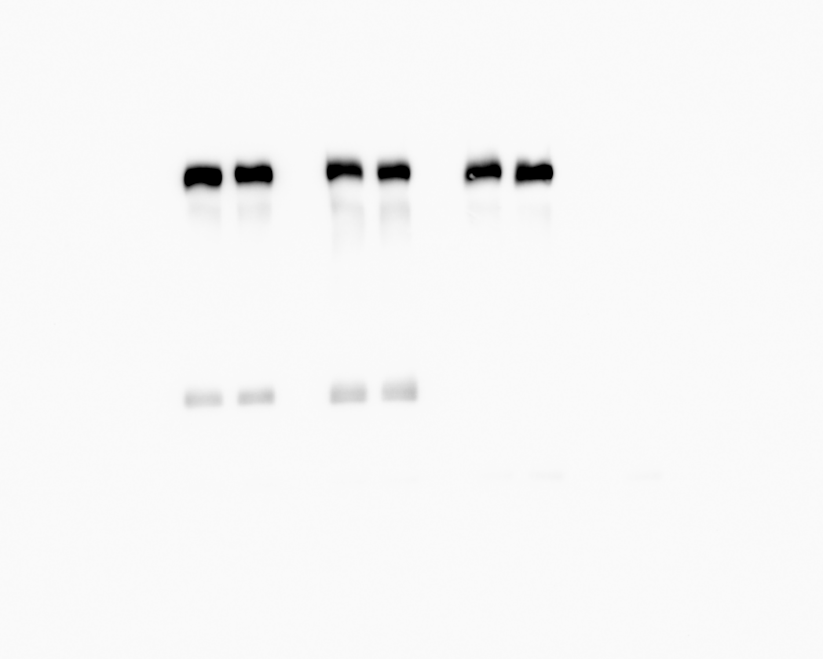


ErbB4: +/+ TUC JMa^-/-^  -/-

TPA: - + - + - + +


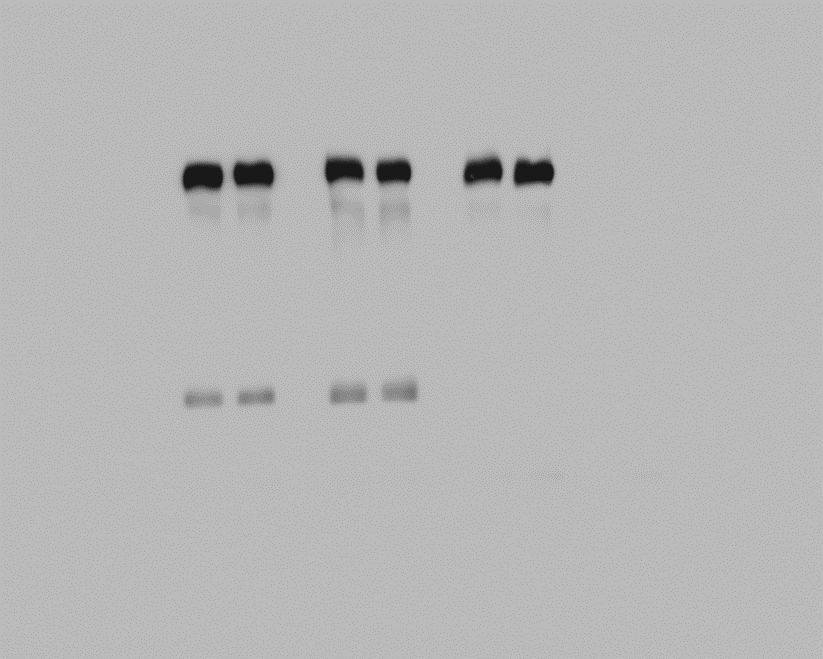


**Fig. S9.** Full image of ErbB4 Western blot on ErbB4 immunoprecipitation from cerebellar lysates to visualize the full-length 180 kD ErbB4 band for Fig 3a. Square shows area cropped for figure. Higher contrast image shown below.


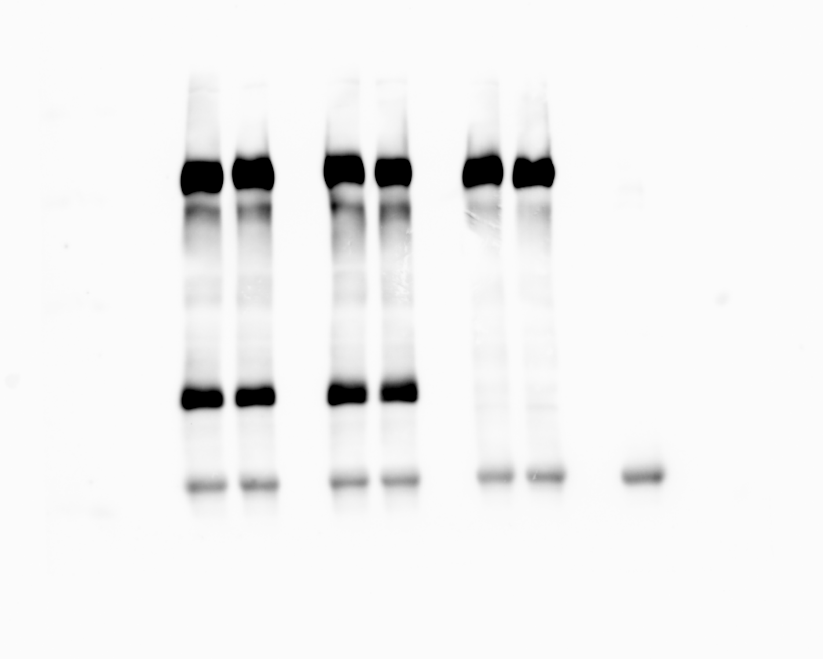


ErbB4: +/+ TUC JMa^-/-^  -/-

TPA: - + - + - + +

**Fig S10.** Full image of ErbB4 Western blot as in SF 8 with longer exposure to visualize the 80 kD E4ICD band for Fig 3a. Square shows area cropped for figure. See above for lower exposure image.


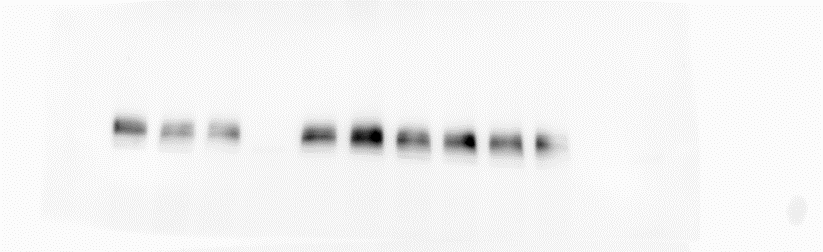


ErbB4: +/+ JMa^-/-^ +/- -/-

ErbB4: +/+ JMa^-/-^ +/- -/-


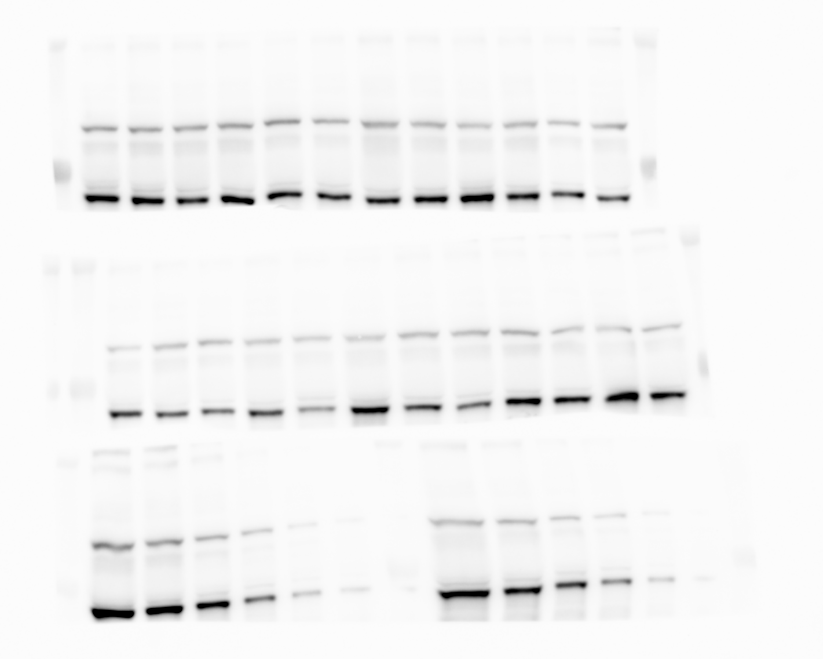


Fig. S11. Full image of ErbB4 (top) and GAPDH (bottom) Western blot on NPC lysates for Fig 3c. The membrane was cut to include relevant molecular weights. Squares shows area cropped for figure.


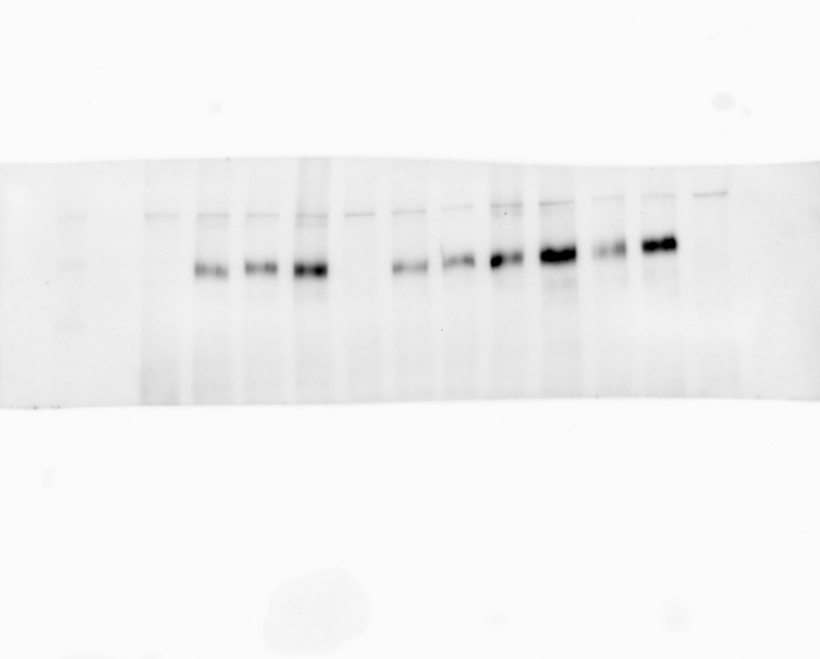


ErbB4: +/+ JMa^-/-^ +/- -/-

Fig. S12. Full image of ErbB4 Western blot of cerebral cortex lysates for Fig 3c. Square shows area cropped for figure.


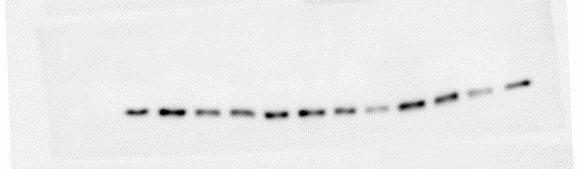


ErbB4: +/+ JMa^-/-^ +/- -/-

Fig. S13. Full image Erbb4 (top) and GAPDH (bottom) Western blot of cerebral cortex lysates for Fig 3c. The membrane was cut to include relevant molecular weights. Square shows area cropped for figure.


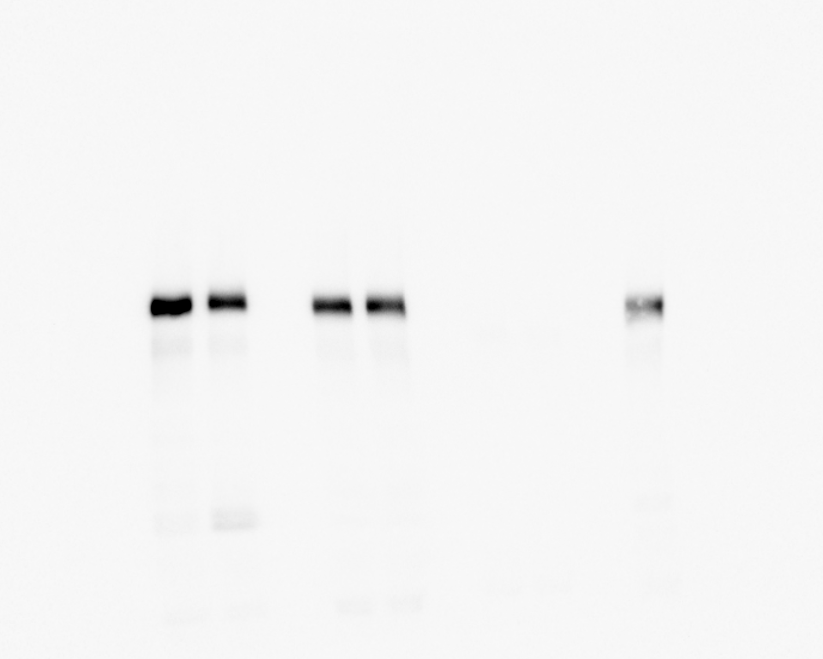


ErbB4: +/+ JMa^-/-^  -/-

TPA: - + - + - +


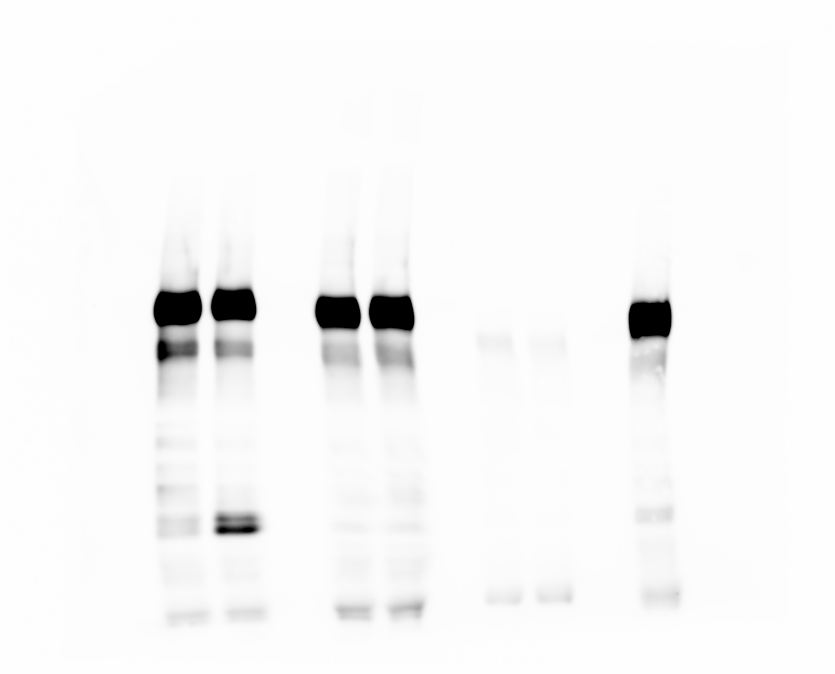


ErbB4: +/+ JMa^-/-^  -/-

TPA: - + - + - +

Fig. S14. Full image of ErbB4 Western blot to visualize the full-length 180 kD ErbB4 (top) and longer exposure to visualize the 80 kD E4ICD band (bottom) for Fig 3d. Square shows area cropped for figure.

FGF: - - + +

NRG1: - + - +


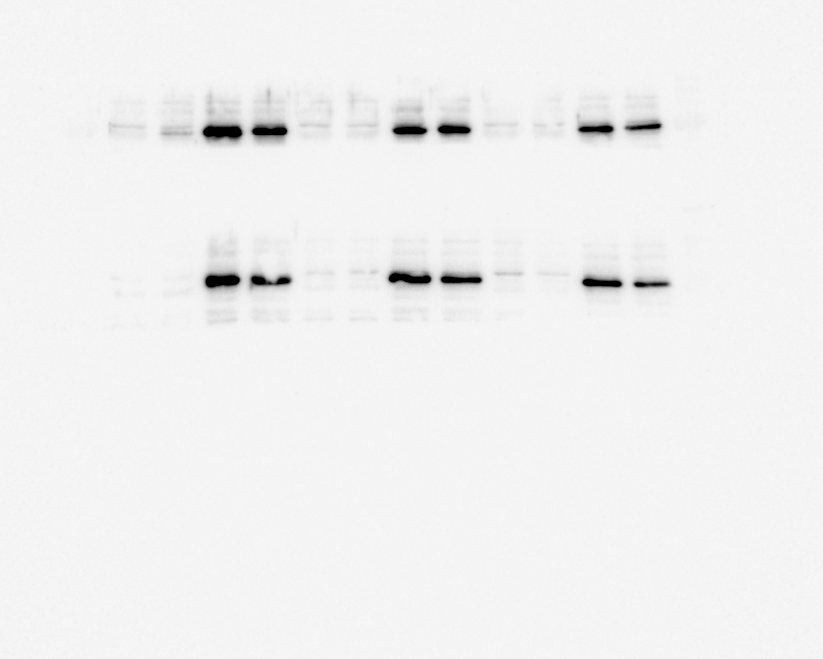


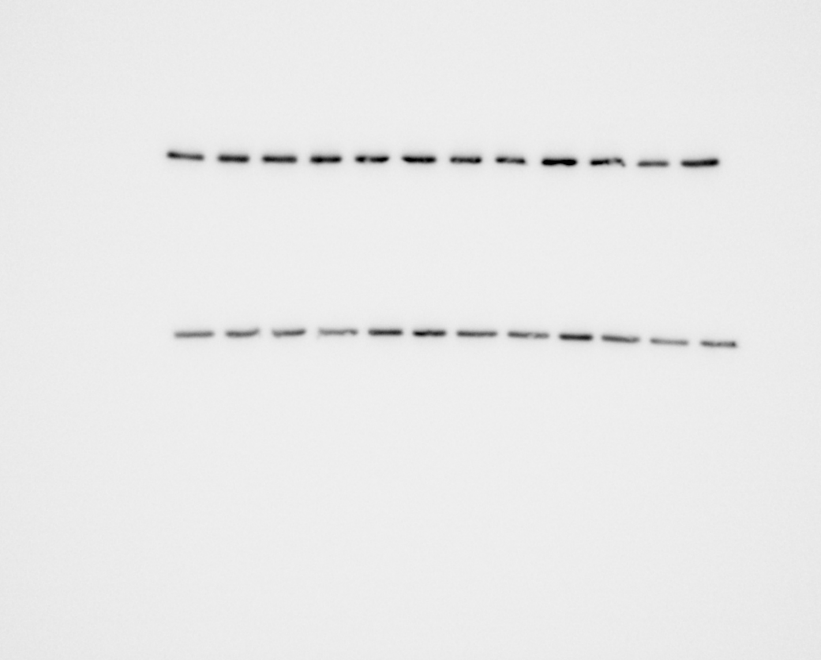


Fig. S15. Full image of GFAP (top) and GAPDH (bottom) Western blot of ErbB4^+/+^ NPCs for Fig 4c. The membrane was cut to include relevant molecular weights. Square shows area cropped for figure.

FGF: - - + +

NRG1: - + - +


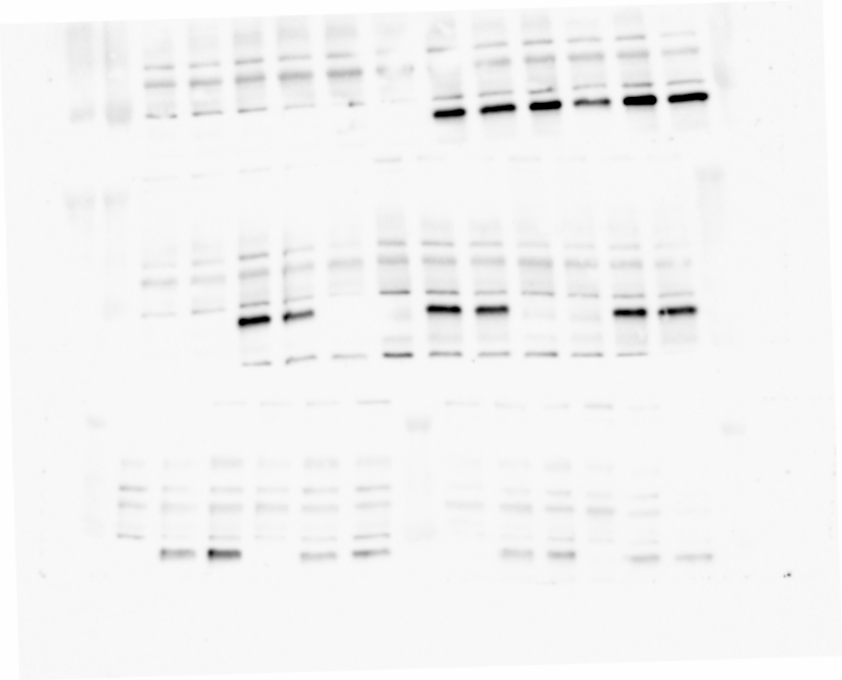


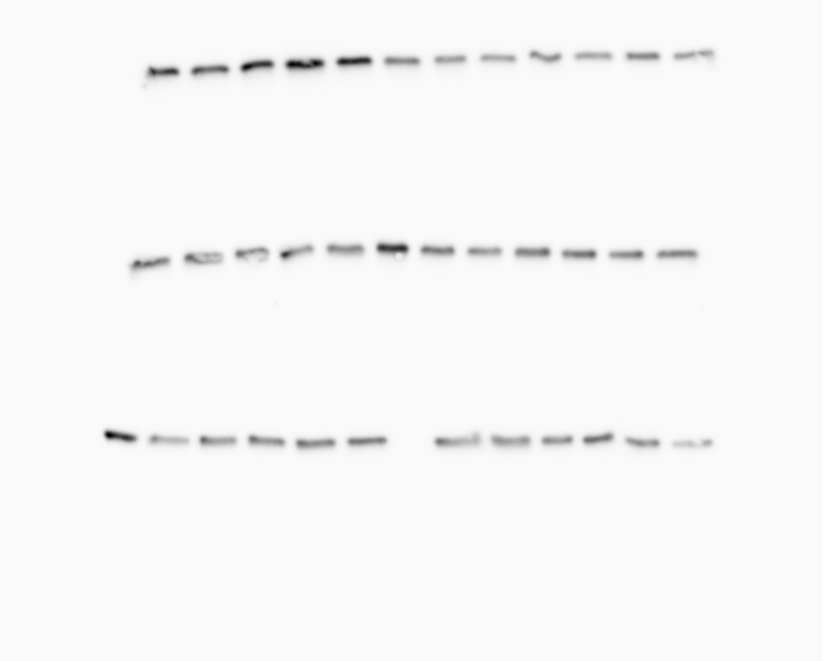


Fig. S16. Full image of GFAP (top) and GAPDH (bottom) Western blot of ErbB4^-/-^ NPCs for Fig 4c. The membrane was cut to include relevant molecular weights. Square shows area cropped for figure.

#
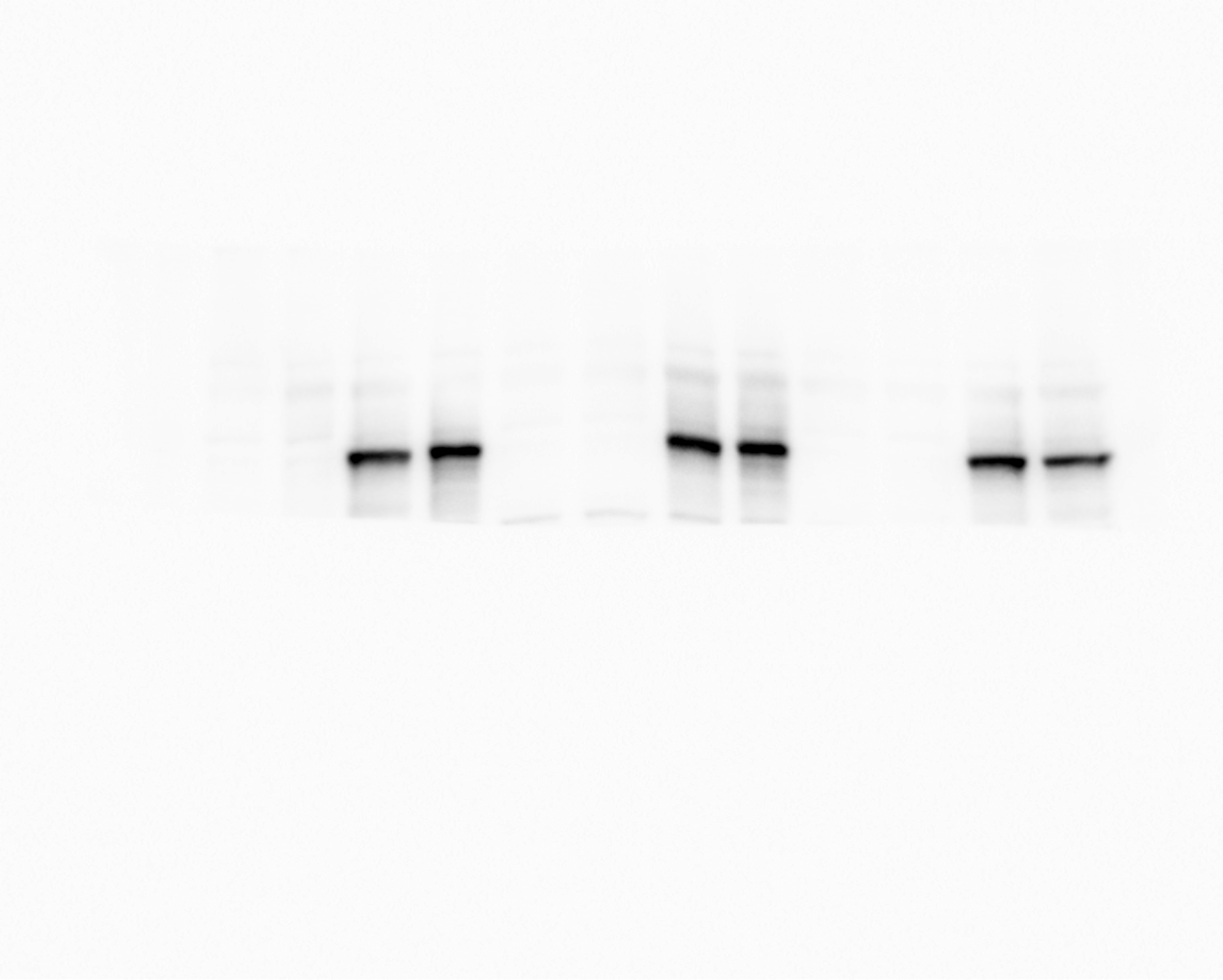


FGF: - - + +

NRG1: - + - +


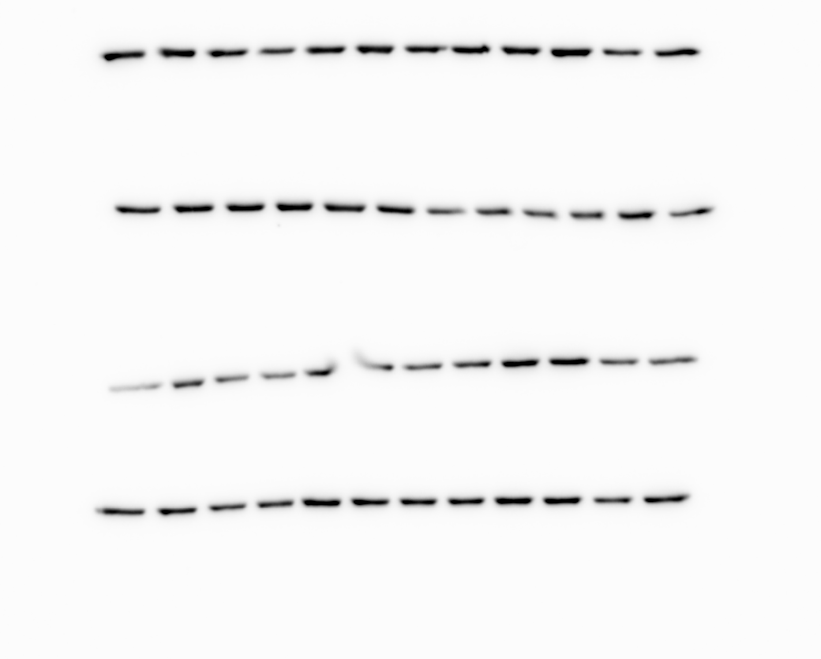


Fig. S17. Full image of GFAP (top) and GAPDH (bottom) Western blot of ErbB4-JMa^-/-^ NPCs for Fig 4c. The membrane was cut to include relevant molecular weights. Square shows area cropped for figure.

FGF: - - + +

NRG1: - + - +


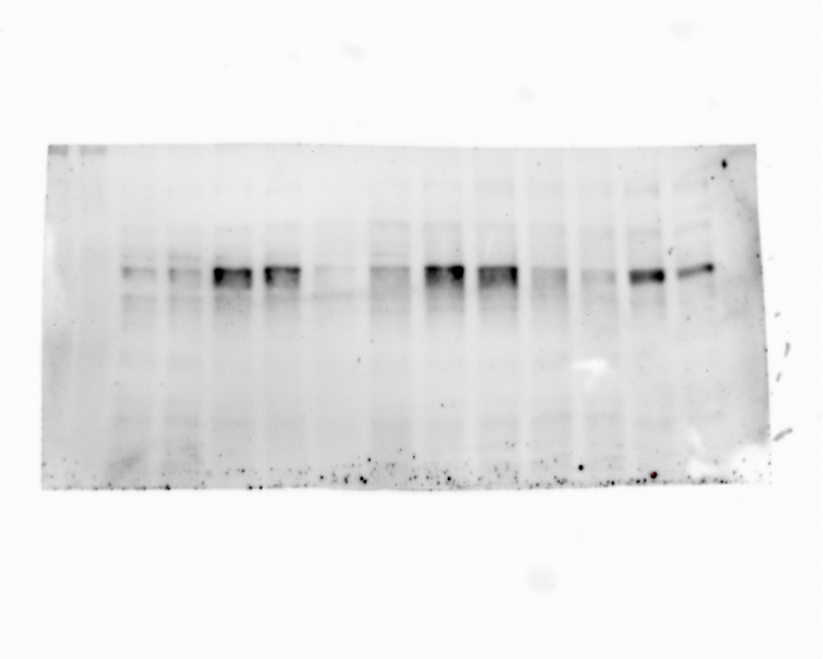


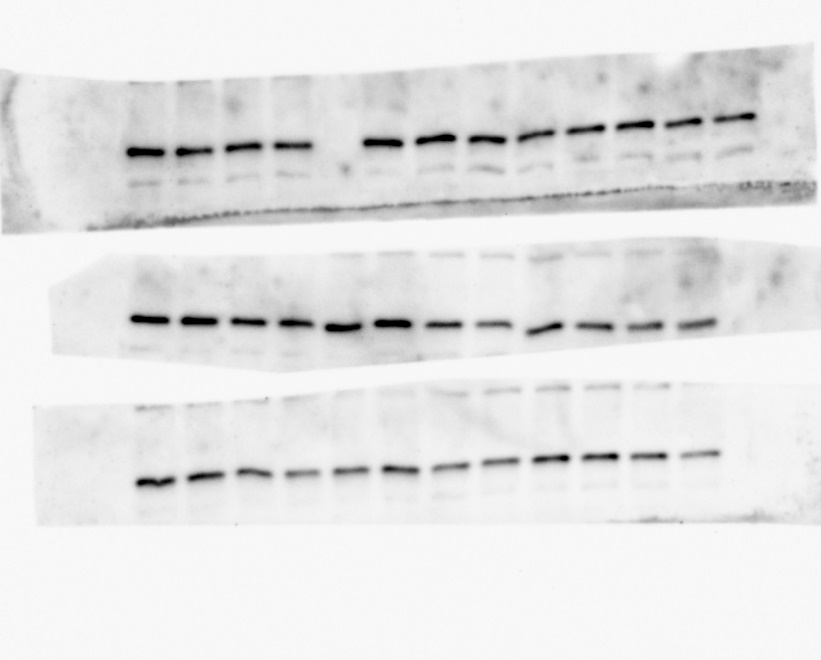


Fig. S18. Full image of GFAP (top) and GAPDH (bottom) Western blot of ErbB4^+/-^ NPCs for Fig 4c. The membrane was cut to include relevant molecular weights. Square shows area cropped for figure.
